# Systems genetics reveals ITIH5 as a key mediator of adipocyte–Endothelial crosstalk

**DOI:** 10.1016/j.molmet.2026.102373

**Published:** 2026-04-21

**Authors:** Mingqi Zhou, Leandro M. Velez, Danica Kwan, Lambda Moses, Casey D. Johnson, Christy M. Nguyen, Lillian Mott, Farah Gamie, Mona Fouladi, Hosung Bae, Amandine Verlande, Douglas Arneson, Paul Petrus, Miklós Péterfy, Andrea Hevener, Remi Buisson, Xia Yang, Lior Pachter, Aldons Jake Lusis, Selma Masri, Dequina A. Nicholas, Cholsoon Jang, Ivan Marazzi, Marcus Seldin

**Affiliations:** 1 Department of Biological Chemistry and Center for Epigenetics and Metabolism, University of California, Irvine, USA; 2 Division of Biology and Biological Engineering, California Institute of Technology, Pasadena, CA, USA; 3 Department of Statistics and Irving Institute of Cancer Dynamics, Columbia University, New York, USA; 4 Department of Pharmacology, University of California Davis, Davis, CA, USA; 5 Department of Medicine, Division of Cardiology, University of California, Los Angeles, USA; 6 Department of Integrative Biology and Physiology, University of California, Los Angeles, Los Angeles, CA, USA; 7 Informatics and Predictive Sciences, Bristol Myers Squibb, San Diego, CA, USA; 8 Department of Medicine (H7), Karolinska Institutet, Stockholm 141 86, Sweden; 9 Department of Biomedical Sciences, Western University of Health Sciences, Pomona, CA, USA; 10 Division of Endocrinology, Diabetes, and Hypertension, Department of Medicine, David Geffen School of Medicine University of California, Los Angeles Los Angeles CA, USA; 11 Department of Molecular Biology and Biochemistry, School of Biological Sciences, University of California Irvine, Irvine CA, USA

**Keywords:** Systems genetics, Adipose cell crosstalk, ITIH5, Adipose-endothelial signaling

## Abstract

Proper adipose tissue homeostasis is essential for systemic metabolic health, and its disruption promotes insulin resistance, inflammation, and cardiometabolic risk. Using unbiased systems genetics analyses in mice and humans identified ITIH5 as a central regulator of adipose homeostasis and whole-body metabolism. Acute administration of recombinant ITIH5 with pan-organ sequencing revealed a local adipose function, suppressing recruitment of circulating immune cells. Consistently, ITIH5 treatment in human endothelial cells reduced leukocyte recruitment. We generated temporally controlled, adipocyte-specific ITIH5 overexpression models in mice, which improved adipose architecture, glucose metabolism under high-fat diet conditions, while consistently reducing left ventricular mass and cardiac output regardless of dietary group. Spatial transcriptomics of adipose tissue showed that elevated ITIH5 signaling to endothelia selectively impairs dendritic cell (DC) and B cell activation pathways. Collectively, these findings identify a mechanism whereby natural genetic variation in an adipocyte-secreted protein modulates endothelial–immune interactions in fat, influencing cardiometabolic homeostasis in a diet-dependent manner.

## Highlights

• Multi-tissue network and genetic centrality identify ITIH5 as a regulator linking adipose coordination to systemic homeostasis.
• Adipocyte-specific AAV models show ITIH5 enhances adipocyte–endothelial signaling and reduces hypertrophy and glucose intolerance.
• Spatial transcriptomics maps adipocyte–endothelial–immune crosstalk, showing ITIH5 suppresses adipose immune activation.
• Integrated spatial and physiological data link ITIH5 immune suppression in adipose to protection against cardiac remodeling.

## 1 Introduction

Adipose tissue plays a central role in maintaining physiologic homeostasis. Altered structure and function of adipose tissue remains a hallmark for most cardiometabolic disorders [[1], [2], [3], [4]]. Hindering comprehensive and detailed characterization of the molecular mechanisms driving cellular composition within this complex tissue has remained limited given that regulation is governed by a variety of genetic and environmental cues. For instance, suppression of inflammation within adipose tissue has been described to significantly change systemic glucose metabolism, where beneficial vs detrimental effects are highly context-specific [[5], [6], [7]]. Depending on the pathways studied, subtypes of immune cells being evaluated or timing of experiment, immune cell functions have been shown to elicit many differing roles in adipose tissue, such as thermogenesis [8,9] and lipid metabolism [10,11].

Systems genetics approaches provides a powerful framework for integrating gene expression, clinical traits, and intercellular coordination to identify key drivers of adipose tissue regulation [12,13]. For example, simultaneous analysis of multiple variables across genetic variation has enabled discovery of many novel genes linked to cardiometabolic dysfunction. Initial surveys of adipose tissue co-expression networks identified genes such as *Lactb* and *Ppm1l*, linked to obesity-related traits [14], which had been missed using classical experimental approaches in single genetic backgrounds. Expanding on this intuition, co-expression analyses widened to incorporate multiple organs with clinical phenotypes and further revealed adipose-liver signaling pathways which impact lipid and glucose metabolism [15]. These approaches stem from a simple intuition in that correlation structure derived from natural variation in gene expression from a population can be used to prioritize central drivers of cell- and organ-signaling [[16], [17], [18]]. When these approaches are paired with experimental systems to define causality, they offer the potential to pinpoint actionable pathways with which to modify disease in a mechanism which operates over a range of genetic backgrounds [[19], [20], [21], [22], [23]].

A healthy immune environment is vital for the adipose tissue to maintain homeostasis [24,25]. Chronic low-grade inflammation in adipose tissue is a hallmark of metabolic dysfunction, contributing to insulin resistance, obesity and type 2 diabetes. Among immune cells, B lymphocytes have emerged as key modulators, exerting both pro- and anti-inflammatory effects depending on metabolic context [26,27]. The ability to signal and retain immune cells is, in part, controlled by cues from local endothelial cells. Endothelial cells also play a central role in adipose inflammation, through cytokine secretion and/or facilitating direct recruitment of immune cells [28,29]. The functions of adipose tissue as an organ requires coordinated actions of primary adipose cells with endothelial lining and consequent recruitment of circulating immune cells to maintain homeostasis, a complex set of interactions which has been challenging to dissect. Despite substantial advances in decoding adipose tissue complexity, critical gaps remain in understanding endothelial cell function and its role in shaping the immune landscape of adipose tissue. Deciphering how endothelial-mediated signaling orchestrates B cell activity and other immune dynamics will be essential to unraveling the context-dependent regulation of adipose tissue homeostasis.

The inter-α-trypsin inhibitor (IαI) family comprises a group of secreted plasma proteins formed from combinations of heavy chains (ITIH1–ITIH5) and the light chain bikunin (encoded by AMBP), which together function as multifunctional protease inhibitors and extracellular matrix (ECM) regulators [30,31]. These proteins circulate in plasma and participate in inflammatory regulation, protease inhibition, and stabilization of extracellular matrices through covalent interactions with hyaluronan (HA) that generate serum-derived hyaluronan-associated protein (SHAP) complexes [[32], [33], [34]]. Transfer of ITI heavy chains onto HA contributes to the formation and stabilization of HA-rich matrices involved in processes such as ovulation, wound repair, and immune cell recruitment [31,32,35]. Individual heavy chain members exhibit specialized roles: ITIH1–3 commonly participate in classical inter-α-inhibitor complexes regulating ECM organization and inflammation, whereas ITIH4 is an abundant plasma glycoprotein associated with acute-phase responses and proteolytic pathways [31]. Increasing evidence also implicates ITIH proteins in diverse disease processes including cancer progression, inflammatory disorders, and cardiometabolic traits [[33], [34], [35], [36]]. Notably, several family member, including ITIH5, have been implicated as regulators of tumor progression and metastasis, where reduced expression correlates with enhanced cell migration and poorer clinical outcomes [37]. Collectively, these studies position the ITIH proteins as multifunctional extracellular regulators linking protease inhibition, matrix organization, and immune signaling in both physiological tissue homeostasis and disease states.

Here, we describe a new mechanism of adipose cellular crosstalk mediated by the secreted protein ITIH5. Population genetics approaches link expression of local adipose signaling of ITIH5 to systemic metabolism and cardiac function in mice and humans. Cell culture and mouse model experimentation demonstrate that ITIH5 signals from mature adipocytes to suppress adipocyte differentiation and blunt the ability of endothelial cells to suppress recruitment of immune cells. Upregulation of the protein via AAV is shown to be sufficient to impact glucose metabolism, respiration and cardiac function. We have generated a novel spatially resolved transcriptomic map of ITIH5 signaling which shows leukocytes recruitment and activation in fat as a chronic mechanism underlying altered adipose architecture and local immune landscape.

## 2 Results

### 2.1 Network of tissue interactions highlights ITIH5 as central to adipose tissue expression and connection to metabolic traits

**Figure 1:**
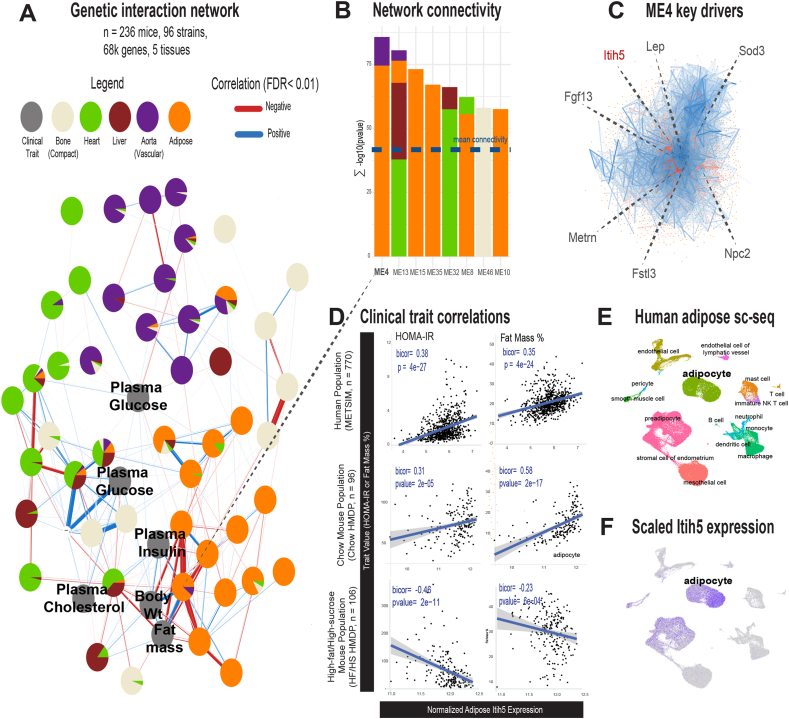
**Genetic network of metabolic interactions prioritizes ITIH5 as a central regulator.** A, Module-based network of tissue gene expression and traits from 96 genetically diverse strains of mice from the HMDP. B, Top modules from A (x-axis) ranked by their interconnectivity throughout the network (y-axis). C, Gene intercorrelation structure of the most interconnected network module (ME4), where the top 7 potential key drivers based on module centrality, secretion annotation and trait correlations are shown. D, Population variation correlations between ITIH5 expression (x-axis) in mouse or humans and traits corresponding to fat mass percentage or HOMA-IR. Bicor shows midweight bicorrelation coefficient and corresponding students regression pvalue. E-F, Cell annotations from human adipose tissue meta-analyses (E), as well as scaled expression of ITIH5 expression (F).

Our first goal was to perform an unbiased survey of potential network interactions within and between metabolic tissues. Therefore, we constructed a module-based network of gene expression data and clinical traits from a collection of 96 genetically diverse mouse strains [38] using WGCNA [39]. While several modules appeared relatively disconnected from the overall network structure, notably from bone, the majority of modules and traits appeared highly interconnected (Figure 1A). Liver showed strong co-correlation with other organs and therefore displayed more membership in shared tissues compared to within-tissue modules. Further, all of the relevant metabolic traits (black nodes) clustered in these network regions of high connectivity with an obvious trend toward correlation with adipose tissue genes (Figure 1A). Of the 48 modules generated from gene expression, the most highly interconnected based on centrality estimates [15,16,22,40] (ME4) consisted of adipose and aortic genes (Figure 1B). This central module was also directly and significantly correlated to plasma insulin, fat mass and body weight (Figure 1A), further validating its relevance for metabolic homeostasis. We next searched for potential drivers of this central module by filtering for within-module centrality, capability of signaling between organs and correlation with metabolic traits in mice and humans (Methods). This analysis highlighted 7 central genes for ME4, most of which are well-established and potent metabolic adipokines (Figure 1C). Among them, *ITIH5* stood out as one gene that is largely uncharacterized in the context of metabolic function. Further, this gene expression was strongly correlated to HOMA-IR and fat mass in mouse [38] and human cohorts [41] (Figure 1D). Interestingly, in the mouse cohort we observed a diet-dependent correlation pattern. In chow-fed mice, the relationship was consistent with that seen in humans, whereas in HFHS-fed mice the expression level of *Itih5* was negatively correlated with HOMA-IR and fat mass (Figure 1D). Mediation analyses using adjusted regressions [[42], [43], [44]] showed that the genetic variance of *ITIH5* expression could explain strong correlations between cardiometabolic traits, as well as trends observed between baseline and follow-up measures in METSIM (Supplemental Fig. 1). In addition, comparisons of 92 genetically-matched strains showed that high-fat feeding significantly induces expression of the gene in adipose tissue (Supplemental Fig. 2).

While little has been described as to the conserved physiologic roles of ITIH5, the gene has been shown to increase in obese humans compared to lean controls [45] and shown to alter gene expression and adipose stem cell differentiation into mature adipocytes [46,47]. In addition, we had previously observed *Itih*5 as an adipose-secreted gene predicted to alter cardiac pathways in genetically diverse mice [19]. In human adipose single-nuclear sequencing [48], *ITIH5* was predominantly expressed in adipose tissue, with the highest levels observed in mature adipocytes (Figure 1E–F) which was validated in an independent cohort of adipose cell fractionation from 80 individuals [49] (Supplemental Fig. 3). These analyses show that *ITIH5* could serve as a central regulator of adipose tissue homeostasis with consequent links to metabolic traits in mice and humans.

### 2.2 ITIH5 acts locally on stromal and endothelial cells to suppress immune cells recruitment

**Figure 2:**
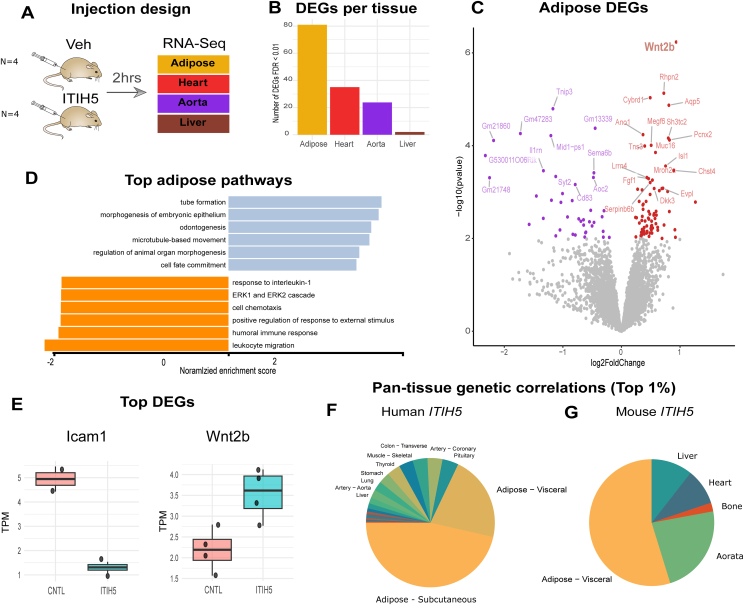
**ITIH5 acts locally in adipose tissue, a process which is suppressed in a HFD.** A, Study design where chow-fed and HFD-fed C57BL/6Nmice were injected with recombinant ITIH5 or GFP, then tissues were subjected to RNA-Sequencing. B, The number of significant (FDR<0.01) genes (y-axis) among various tissues (x-axis), where adipose is the highest. C-D, Differentially-expressed genes within adipose tissue shown as a volcano plot (C), associated enrichment of significant upregulated (blue) and downregulated (orange) pathways (D). E, boxplots of normalized TPM for Icam1 and Wnt2b in adipose from the rITIH5 injection model, showing suppression of Icam1 and induction of Wnt2b.F-G, The proportion of genes per tissue among the top 1% correlated to ITIH5 in human (F) or mice (G) fat. N = 4 mice per group.

Given that ITIH5 has been implicated in several physiologic roles, we next asked what direct organs and processes are engaged by acute ITIH5 actions. Therefore, mice were acutely (2 h) administered either vehicle (Veh) or recombinant ITIH5 protein (0.1ug/gram body weight) and key tissues were subjected to RNA-sequencing (Figure 2A). Quantification of the number of differentially expressed genes (DEGs) showed that the largest response to ITIH5 occurred in visceral adipose tissue (Figure 2B). Specifically, the protein upregulated pathways related to Wnt/developmental signaling and reduced immune cell recruitment and response (Figure 2C–E). The autocrine/paracrine signaling of ITIH5 observed in mice also appeared to translate to humans. Comparison of within-vs between- organ signaling based on genetic correlation [19,40,50] structures showed that genes strongly correlating with *ITIH5* expression were more enriched in adipose than in other tissues in humans (Figure 2F) and mice (Figure 2G). In contrast to this physiologic signaling mechanism, feeding mice a HF/HS diet prior to injection resulted in a minimal number of differentially expressed genes in adipose and showed a stronger degree of induction in heart (Supplemental Fig. 4). These data show that acute responses to ITIH5 are dominated by local autocrine/paracrine signaling in adipose tissue (visceral), a process which is actively suppressed by a high-fat diet.

**Figure 3:**
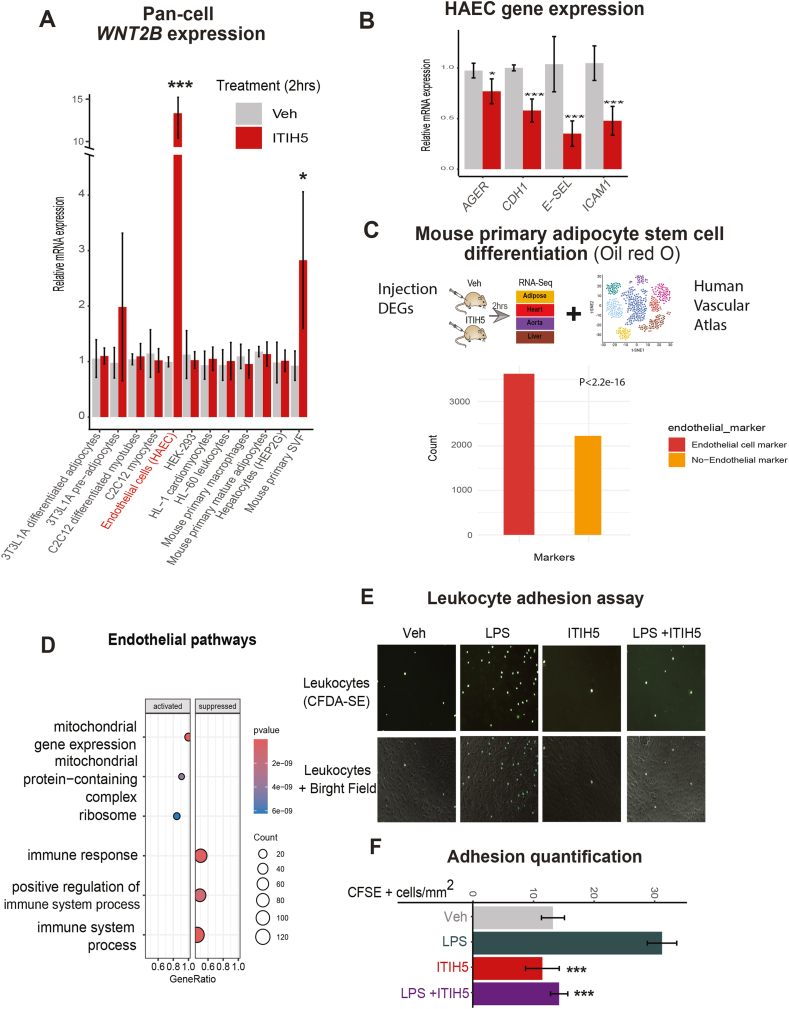
**ITIH5 suppresses adipocyte differentiation and endothelial cell recruitment of immune cells.** A, qPCR analysis for relative gene expression levels of *WNT2B* (y-axis) among multiple primary or immortalized cells (x-axis) when treated with Veh or ITIH5 (n = 6). B, Gene expression levels for antigen presenting proteins or adhesion molecules in HAECs when cells treated with Veh or ITIH5 (n = 6). Comparisons for both A and B were made between veh and ITIH5 treatment using a students t-test and corresponding pvalues shown are: ∗ = P < 0.05, ∗∗∗P < 0.001. C, Overlap of differentially expressed genes from the rITIH5 mouse injection model with human vascular single-cell transcriptomic data, highlighting genes predominantly expressed in endothelial cells. D, Gene Set Enrichment Analysis of endothelial-enriched genes reveals downregulation of immune response–related pathways. E-F, Leukocyte adhesion assay representative images (E) and quantification (F), where HAECs were pretreated with ITIH5 (0.1ug/mL), LPS (5 ng/mL) or both then CFDA-SE-labelled HL60 leukocytes were added and imaged for adherence (n = 20). A = pvalue<0.01 vs veh; b = pvalue<0.005 vs veh and c = pvalue<0.005 vs LPS condition using a students t-test. Students t-tests were used for comparisons following confirmation of normality for each group using Shapiro–Wilk tests.

To pinpoint the target cell types on which ITIH5 acts, diverse primary or immortalized cell lines were treated with veh or ITIH5 and assayed for expression of *Wnt2b* (Figure 3A), the gene most strongly induced in mice (Figure 2C,E). Consistent with known roles in developmental effects on adipocytes [46,47], induction of *Wnt2b* was modestly localized to stromal fractions, pre-adipocytes (Figure 3A). We note that pharmacologic inhibition of Wnt signaling via IWP2 [51] failed to blunt the ability of ITIH5 in suppressing adipocyte maturation and lipid accumulation via Oil Red O (Supplemental Fig. 5). Surprisingly, *WNT2B* showed the largest degree of induction in human aortic endothelial cells (HAEC) (Figure 3A). Next, we wanted to explore the new roles of the protein in acting on endothelial cells beyond the large induction of *WNT2B* (Figure 3A). Consistent with acute administration of the protein driving suppression of pathways related to immune cell recruitment (Figure 2D), HAECs treated with ITIH5 showed a significant reduction in expression of regulators of immune cell recruitment and adhesion (Figure 3B). In further support of endothelial cells as mediators of the primary physiologic responses to ITIH5, RNA-seq DEGs from recombinant protein injections were intersected with the Human Vascular Cell Atlas [52]. This analysis showed that over half of the differentially expressed genes from injected mice were predominantly found in endothelial cells (Figure 3C). Gene Set Enrichment Analysis (GSEA) further demonstrated that these endothelial-enriched genes recapitulated a downregulation of immune response pathways (Figure 3D), a pattern not observed in non-endothelial cells (Supplemental Fig. 6). To verify changes in gene expression at the functional level, we adopted an assay to culture endothelial monolayers and monitor capacity of leukocyte adhesion and recruitment [53]. Endothelial cells treated with an inflammatory stimulus (LPS) increased leukocyte recruitment to endothelia, where ITIH5 protein alone blunted this adhesion of leukocytes (Figure 3E–F). Collectively, these data provide a new role for ITIH5 in signaling from mature adipocytes to suppress recruitment of immune cells via endothelia.

### 2.3 Chronic upregulation of ITIH5 alters adipose tissue architecture which is exacerbated by a high-fat diet

**Figure 4:**
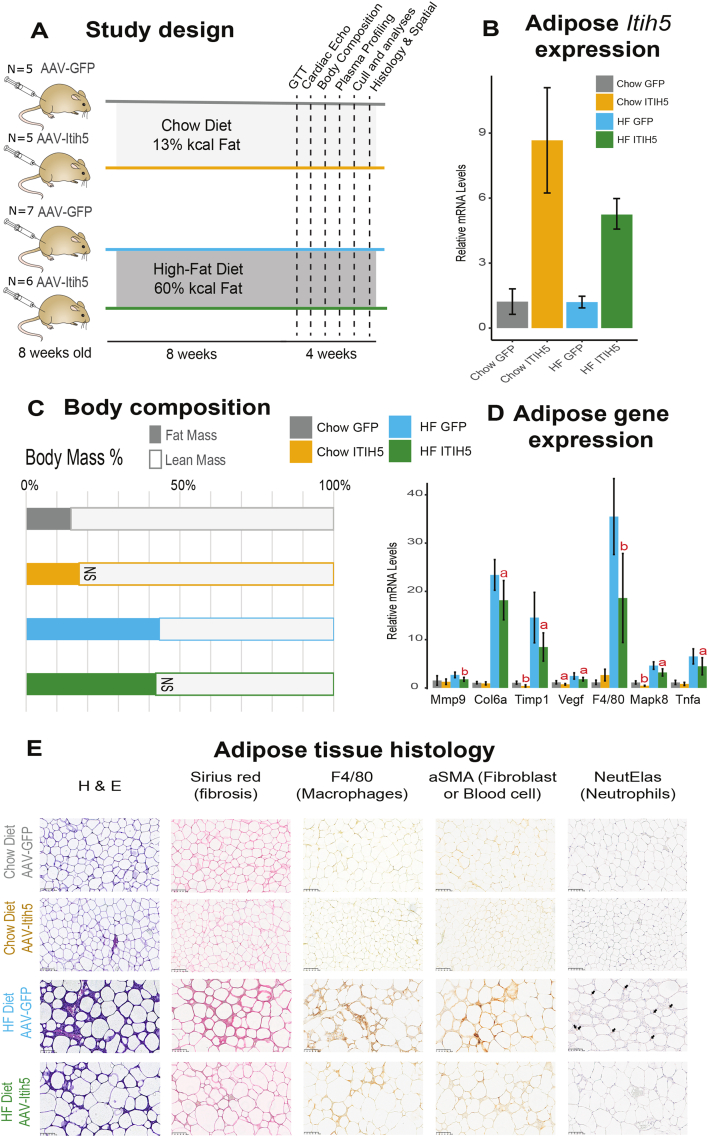
**Chronic ITIH5 expression prevents adipose tissue changes associated with a HFD.** A, Study design, where adiponectin-AAV was administered to 8-week-old C57BL/6N male mice which was followed by either normal chow or high-fat diet and various metabolic assays. B, Magnitude of Itih5 mRNA expression in visceral adipose tissue following study conclusion. Bars show mean +/− 95% CI, n = 9–11. C, Bar charts showing mean values for each AAV group of NMR mass. D, qPCR analysis of indicated genes and AAV groups in visceral adipose tissue. Bars show mean +/− 95% CI, n = 9–11. Statistical differences assessed using students t-test (two-way). a = pvalue<0.01, b = pvalue<0.005. E, Representative visceral adipose tissue images for crude histology (H&E), Sirius red (collagen fibers), F4/80 antigen, alpha-smooth muscle actin or neutrophil elastase. Magnification at 10x, 100um bars shown in bottom right corner. N = 5 mice for chow AAV-GFP, N = 5 mice for chow AAV-ITIH5, N = 7 mice for HFD AAV-GFP, N = 6 mice for HFD AAV-ITIH5. Comparisons between two groups (AAV-GFP vs AAV-ITIH5) were made using students t-tests following confirmation of normality for each group using Shapiro–Wilk tests.

To evaluate the global consequences of sustained ITIH5 upregulation from adipocytes, adult mice were administered adeno-associated virus 8 (AAV8) containing either control (AAV-GFP) or Itih5 (AAV-Itih5) under an adiponectin promoter, then fed a normal chow or HFD (Figure 4A). We acknowledge that adiponectin expression is not restricted to mature adipocytes alone [54], this system was developed to enable post-developmental tuning of ITIH5 expression. While a ∼5–8fold increase in *Itih5* (Figure 4B) had no effect on body weight fat or lean mass (Figure 4C, Supplemental Fig. 7), the protein significantly altered visceral adipose tissue gene expression and morphology. Specifically, overexpression of *ITIH5* reduced the expression of proinflammatory genes (Figure 4D), macrophage, neutrophil abundances, as well as fibrotic staining (Figure 4E, Supplemental Fig. 8), notably under HFD settings. The actions of ITIH5 in modulating adipose tissue physiology appeared relatively specific, as no similar changes were observed in other metabolic tissues such as liver and skeletal muscle (Supplemental Fig. 9).

ITIH5 belongs to a family of trypsin protease inhibitors and other members that have been described to modify ECM remodeling through a covalent linkage with bikunin [55]. In light of these observations, bikunin banding patterns were immunoblotted in all AAV groups in serum and adipose tissue. The proportion of bikunin bound to an ITIH (evident by size shifts) was not different between any groups (Supplemental Fig. 10), indicating that linkage and ECM remodeling via bikunin was not playing a role in these models where metabolic effects were observed. These results indicate that ECM-remodeling via bikunin does not play a role in the observed effects of adipose ITIH5 in mice. These data show that chronic upregulation of ITIH5 alters fat architecture and prevents fibrosis and inflammation during a high-fat diet regimen through pathways distinct from other inter-alpha trypsin inhibitor family members.

### 2.4 Spatial transcriptomic map of ITIH5 cellular responses shows significant reductions in B-cell actions in response to changes in recruitment and differentiation changes

**Figure 5:**
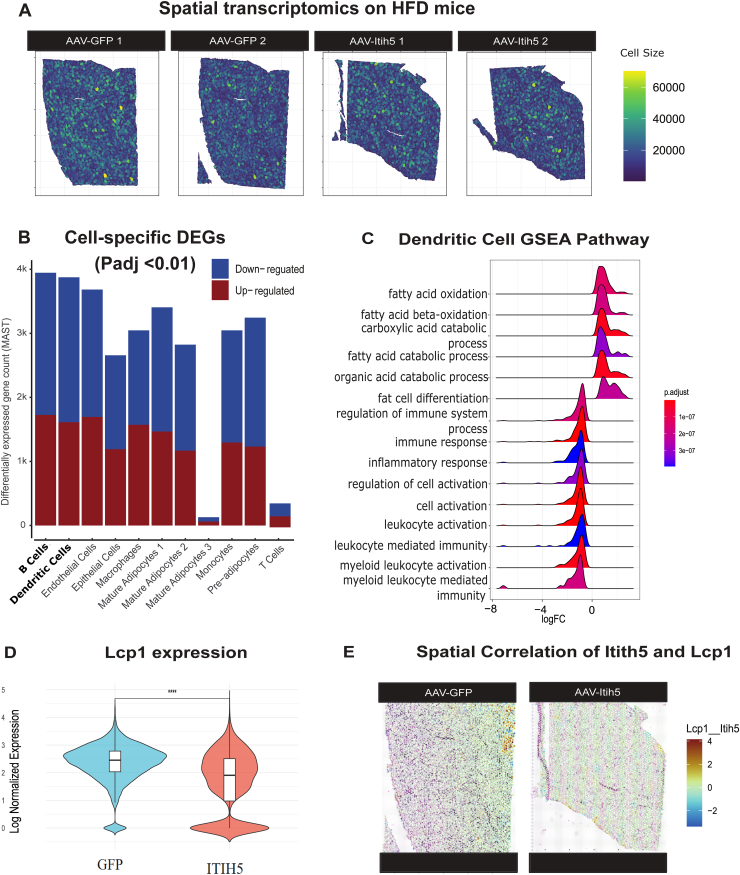
**Spatial transcriptomic analysis shows dendritic cell activation is suppressed by chronic upregulation of ITIH5.** A, Spatial transcriptomic visualization of inferred cell sizes across tissue sections from AAV-GFP (control) and AAV-Itih5-treated mice. B, Differentially expressed genes (DEGs) across major cell types, comparing AAV-Itih5 and AAV-GFP groups. dendritic cells exhibited a great number of DEGs, with a substantial proportion being downregulated (blue) or upregulated (red). C, Gene set enrichment analysis (GSEA) of dendritic cells highlighting the suppression of immune-related pathways, including immune response, inflammation response, myeloid leukocytes activation and meditate immunity. D-E, Violin plots depicting the absolute expression levels of immune cell markers *Lcp1*. *Lcp1* expression showed a marked reduction in AAV-Itih5-treated mice compared to controls. E, Spatial correlation analysis between *Itih5* and the pan-leukocytes marker *Lcp1* using Lee's spatial correlation statistic.

Given the complex nature of ITIH5 interactions and potential implications for adipose tissue homeostasis, we performed Visium spatial transcriptomics on HFD AAV-overexpression models. Consistent with the effects on tissue remodeling, we observed differences in cell size distribution between AAV-GFP and AAV-ITIH5 adipose compartments (Figure 5A). Differential expression analysis across cell clusters re-confirmed stronger responses in endothelial cells compared to adipocytes (Figure 5B); however, the largest number of significant DEGs was observed in B cells and followed by the dendritic cells (Figure 5B). Gene set enrichment analyses show that chronic ITIH5 upregulation suppresses activation of core dendritic call function, upregulated the fatty acid oxidation and blunt the immune and inflammation response (Figure 5C). Also impaired the B cell functions such as antibody production and fatty acid metabolism (Supplement Fig 11), where all cell-specific DEGs are provided in Supplemental Table 1. Expression of pan-leukocytes marker gene *Lcp1* and immunoglobulin marker genes *Igkc* and *Ighg2b* showed significant differences between groups exclusively in dendritic cells and B cells (Figure 5D–E and Supplement Figure 11). Spatial analyses enabled us to pinpoint relevant proximal relationships among gene expression patterns. For example, despite modest expression levels of *Itih5* and *Lcp1* in the GFP AAV mice, we observed strong spatial co-clustering of the immune responses facilitating gene *Lcp1* depending on *Itih5* expression. Specifically, coexpresion between the two genes were inversely correlated in GFP settings, which was disrupted due to the marked overexpression in the AAV-Itih5 groups (Figure 5E). These data support a role for the protein in modulating the adipose immune landscape, specifically through the suppression of dendritic cell activation. Our results demonstrate that chronic ITIH5 elevation significantly reduces the expression of *Lcp1* (Figure 5D), a critical mediator of actin-dependent DC migration and antigen presentation. This downregulation, combined with the loss of spatial co-clustering, suggests that ITIH5 overexpression effectively blunts core dendritic cell functions, thereby dismantling the homeostatic immune hubs required to regulate local leukocytes responses and inflammation. In addition to *Itih5* with *Lcp1*, spatial correlations between endothelial and B cell markers showed robust spatial co-clustering patterns which were significantly reduced following chronic adipocyte *Itih5* overexpression (Supplemental Fig. 12). These data provide a spatial atlas of cell type expression changes in response to chronic elevation of ITIH5, highlighting dendritic cell activity as the largest responsive actions to chronic upregulation of adipocyte-endothelial crosstalk.

### 2.5 ITIH5 modulates glucose homeostasis and cardiac function in a diet-dependent manner

**Figure 6:**
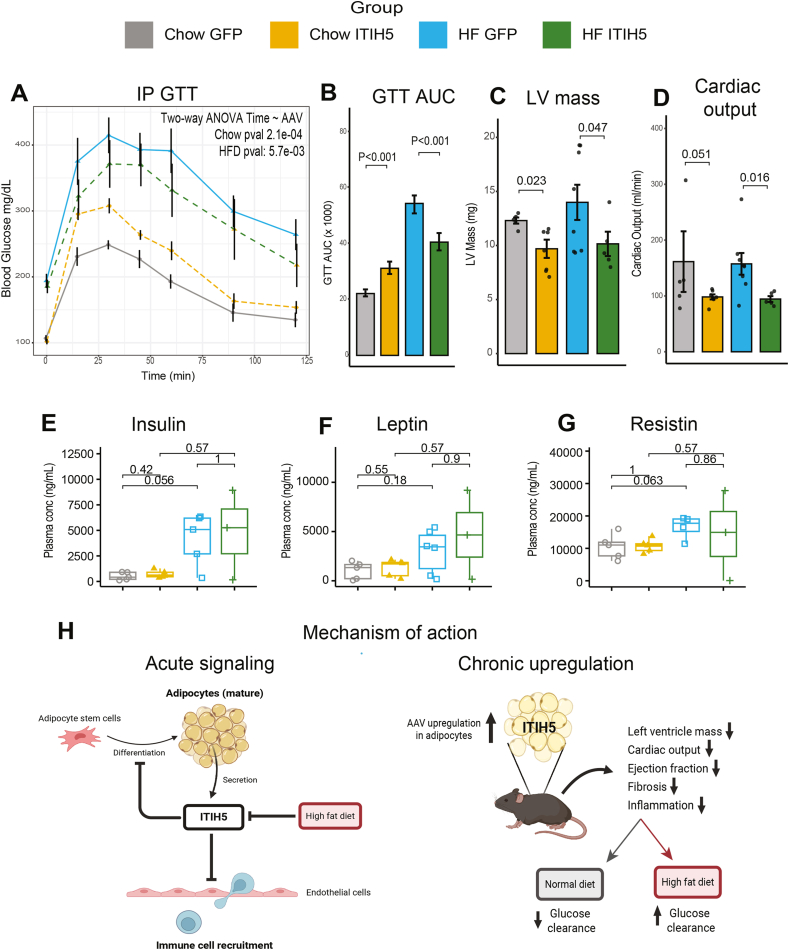
**Chronic ITIH5 over-expression drives whole-body cardiometabolic responses to a HFD.** A, Intraperitoneal glucose tolerance test on indicated diet or AAV groups. Two-way ANOVA Pvalue for Itih5 vs GFP within each dietary condition. We note that the ANOVA for all groups (ex. interaction between diet and AAV, as opposed to interaction between AAV and time) achieved a Pvalue = 0.14, likely due to opposing effects fo AAV-ITIH5 in the two diets. B, AUC quantification for GTT. C-D, Echocardiographic parameters assayed for AAV groups and diets quantified for Left Ventricular Mass (C) or Cardiac Output (D) E-G, Circulating hormone concentrations in AAV mice quantifying Insulin (E), Leptin (F) or Resistin (G). H, Schematic illustration of the proposed role of ITIH5 in adipose and vascular function. ITIH5, secreted by mature adipocytes, inhibits adipocyte differentiation and suppresses endothelial cell-mediated immune cell recruitment. Systemically, ITIH5 exhibits diet-dependent effects on metabolic regulation, enhancing glucose uptake under HFD conditions while reducing it in chow-fed mice. Despite these metabolic differences, ITIH5 consistently impairs cardiac structure and function, as evidenced by reduced left ventricular mass, cardiac contractility, and ejection fraction under both dietary conditions. Bars show mean +/− standard deviation, n = 9–11. Pvalues calculated using two-way students t-test between GFP and Itih5 groups within each diet. a = pvalue<0.01, b = pvalue<0.005.

We next evaluated the physiologic consequences of local effects of ITIH5 observed in adipose tissue. When subjected to a glucose tolerance test (GTT), chow-fed AAV-Itih5 mice showed a decreased ability to clear glucose from circulation; however, HFD AAV-Itih5 mice had enhanced glucose clearance compared to GFP controls (Figure 6A). These data are consistent with suppression of adipose tissue immune function eliciting a protective role under normal conditions, but exacerbates diet-induced metabolic dysfunction [35,36,37]. Regardless of dietary regiment, however, echocardiographic assessment showed that left ventricular mass (Figure 6B) and consequent cardiac output (Figure 6C) were significantly reduced in both ITIH5 over-expression groups. Similar structure-functions changes were observed in other cardiac measures (Supplemental Table 2). These metabolic and cardiac responses to ITIH5 occurred independent of significant changes in plasma insulin (Figure 6D), leptin (Figure 6E), resistin (Figure 6F) or several other key hormones (Supplemental Fig. 13). These results demonstrate that *Itih5* alters adipose morphology and glucose homeostasis in a diet-dependent manner; however, regardless of environmental influence, suppresses cardiac output. In addition, shifts in cardiac and metabolic functions being driven by ITIH5 occurred independently of classical hormones such as insulin, leptin or GLP1, suggesting that local adipose immune cell activation influences systemic physiology through alternative mechanisms. We therefore propose a two-part mechanism of action for ITIH5 as a central mediator of adipose tissue homeostasis (Figure 6G). Acutely, ITIH5 secreted by mature adipocytes inhibits adipocyte differentiation and acts on local endothelial cells to suppress immune cell recruitment. Chronically, sustained activation of this signaling circuit leads to blunted immune activation and fibrosis in fat. This chronic remodeling drives diet-specific changes in systemic glucose metabolism, while consistently reducing cardiac output and left ventricular mass regardless of the dietary context.

## 3 Discussion

In the present study, we uncover the adipocyte-specific factor ITIH5 as a local regulator of adipocyte differentiation and endothelial cell function, resulting in changes in metabolic and cardiac homeostasis. While this study is the first to evaluate the whole-body role of ITIH5, many questions remain as to how local adipose signaling engaged by ITIH5 leads to alterations in glucose metabolism and cardiac function. We characterized a landscape of cellular changes in adipose tissue, including direct signaling to preadipocytes and endothelial cells and resulting reduction of leukocytes recruitment. Notably, our spatial and transcriptomic analyses revealed that DCs serve as a primary immunological node in this process. While our initial analysis identified a B-cell signature, subsequent prioritization of the DC population uncovered a more biologically plausible driver of the observed phenotype. Whether effects on adipogenesis, endothelial cell actions, immune cell composition or a combination of all actions underlies systemic changes in metabolism remain open. Clearly, altering any of these 3 processes in adipose tissue is sufficient to lead to changes in whole-body metabolism. Other ITIH family members have been shown to modify ECM remodeling through a covalent linkage with bikunin [55]; however, we showed no evidence for these interactions with ITIH5. More focused characterization of the mechanisms of ITIH5 action will help to clarify this question, notably which receptor and canonical cellular signaling pathways ITIH5 acts on in order to elucidate cell-specific functions.

Our data implicates Wnt signaling in the mechanisms of ITIH5 actions. Several pharmacologic compounds and genetic knockouts of Wnt have already been demonstrated to modify systemic glucose sensitivity through adipose tissue, supporting this hypothesis [[56], [57], [58], [59]]. While prior studies have shown that Wnt5a-mediated non-canonical WNT signaling reduces fibrosis and increases lipid accumulation, they also report an upregulation of inflammation [60]. While our data focused on *Wnt2b* and differing signaling pathways, we observed contrasting observations that suggest upregulation reduces endothelial cell recruitment capacity as a core feature of ITIH5 action. Importantly, Wnt signaling has been shown to play crucial roles in adipogenesis. For example, WNT signaling has been shown to inhibit de novo adipogenesis from stem cell populations as well as other relevant cell types such as osteoblasts [[61], [62], [63], [64]]. Because this inhibition primarily targets early stem cell commitment rather than the expansion of existing fat depots, manipulating this pathway in our adult cohort was likely insufficient to significantly alter total body weight. Identification of the receptor and canonical signaling pathways whereby ITIH5 acts to exert its effects will contextualize specific mechanisms linking the protein to actions on preadipocytes and endothelial cells.

Beyond these direct endothelial actions, our data positions DCs as a primary immunological node in ITIH5-mediated homeostasis. While macrophages are traditionally viewed as the main inflammatory effectors in obesity, recent evidence establishes that conventional DCs accumulate early in obese adipose tissue to orchestrate subsequent leukocyte infiltration [65,66]. Dendritic cells have been recently described as emerging drivers of adipose tissue function and inflammation associated with metabolic diseases [67]. GSEA analysis of DC population demonstrated a significant downregulation of cell activation pathways upon ITIH5 overexpression, corresponding directly to the suppressed leukocyte adhesion observed in the functional assays. This ITIH5-mediated suppression is driven by a structural reorganization of the adipose microenvironment. Using Local Lee's statistics to map the pan-leukocyte marker *Lcp1*, ITIH5 overexpression disrupts the typical proximity between leukocytes and mature adipocytes. Control tissues exhibited significant spatial leukocyte clustering, characteristic of obese inflammation, which was absent in the treated group. Concurrently, our transcriptomic data revealed that ITIH5 induces metabolic reprogramming within these DCs, specifically upregulating fatty acid oxidation. Because DC maturation relies heavily on glycolytic shifts to meet inflammatory energy demands, this upregulated process acts as a metabolic restraint, maintaining the DCs in a tolerogenic state even under high-fat diet conditions.

Additionally, understanding why the protein exerts such significant cell-specific effects and alters adipose histology while no differences are observed in total fat mass remains an important question. The only report of genetic mutation of ITIH5 showed that removing the protein enhances fat mass expansion [47]. Our findings build on recent observations from whole-body knockout models [47,68] and extend actions of ITIH5 to a new adipocyte-endothelial signaling circuit. While Sessler et al. reported that Itih5 deficiency exacerbates inflammation and fat mass, our adipocyte-specific AAV model administered to adult mice similarly suppressed inflammation; however, showed no effects on total body weight or fat mass. This discrepancy could be due to the temporal nature of the interventions. Germline deletion impacts adipose development [47], whereas ITIH5 upregulation in mature mice has bypassed a crucial check-point in adipocyte differentiation. An alternative explanation for this discrepancy is that, while deletion of the gene accelerates fat mass expansion similar to that of obesifying conditions, additional endogenous mechanisms exist to prevent persistent suppression of differentiation from upregulation of the gene. Consistent with this notion, our data shows that in conditions of accelerated fat mass growth from a high-fat diet, causes adipose-specific resistance to ITIH5 actions (Supplemental Fig. 4). Finally, we acknowledge that the degree of upregulation allowed by the AAV system used in this study could not reach a high enough threshold to globally alter fat mass. In this light, the dose-dependent effects of ITIH5 have been shown as important for its impacts on ASCs as well [68].

Distinguishing the relative contributions of adipocyte differentiation or endothelial recruitment of immune cells to systemic glucose metabolism and cardiac function will assist in understanding the conserved roles of the protein. Both alterations of endothelial cell recruitment and adipocyte differentiation have been widely described to influence systemic physiology where our spatial transcriptomic analyses suggest endothelial recruitment of leukocytes as a key role of the protein. Specifically, using Local Lee's statistics to map the pan-leukocyte marker Lcp1, we found that Itih5 overexpression disrupts the typical proximity between leukocytes and mature adipocytes. In the control state, Lcp1+ cells, including macrophages, neutrophils, and DCs, showed significant spatial clustering around fat cells, indicating an active inflammatory environment. Extending these local actions to their roles in systemic physiology will assist in deconvoluting mechanisms of inter-organ signaling in cardiometabolic disease. ITIH5 may circulate to the aorta and heart, where it interacts with endothelial cells and alters crosstalk with cardiomyocytes [69]. This interaction could promote oxidative stress and contribute to the increased mitochondrial activity we observed in the heart and aorta. For example, our data show that ketone body levels differed upon *Itih5* over-expression in the heart during a high-fat diet where changes were more pronounced. Whether altered leukocytes recruitment in adipose tissue is sufficient to alter hydroxybutyrate usage in heart has yet to be determined.

One key remaining question is disentangling the contributions of ITIH5 actions on whole-body metabolism through cell- or organ-specific functions. While we focus on mechanisms of local paracrine signaling in adipose tissue, we note that the protein was still observed to alter gene expression in other tissues, notably the heart. Clearly, vascular endothelial cells have been demonstrated to play critical roles in whole-body metabolism, insulin resistance and cardiac function [70,71]. Given that the heart is a highly-vascularized organ and that altered endothelial cell physiology drives changes in cardiac output [72], it is also plausible that the physiological changes observed in AAV models could be due to endocrine actions of ITIH5. Understanding the organ-specific contributions to these effects will be critical in understanding how endothelial cells contribute to metabolic homeostasis from discrete tissues.

Our data shows that the role of the protein shifts depending on dietary context. Specifically, effects are more robust, and in the case of glucose clearance even opposite, when mice were fed a high-fat diet. The consistent reduction in cardiac output observed in both chow and HFD mice overexpressing ITIH5 suggests that the effect on cardiac function is not solely mediated by nutrient or hormonal status but rather by a structural or inflammatory reprogramming of adipose tissue. We propose that ITIH5-driven suppression of leukocyte adhesion and transmigration into adipose tissue, largely via endothelial cell remodeling, leads to a dampened immune response which protects against diet-induced metabolic effects, but fail to maintain proper production or release of cardioprotective adipose-derived factors. This could include reduced expression of proangiogenic or cardiotonic adipokines such as VEGF-A [73,74], apelin [75], or high-molecular-weight adiponectin [76] that are known to act on cardiac endothelium and myocytes. Alternatively, the dampening of low-grade adipose inflammation may reduce the basal production of extracellular vesicles or metabolites such as lipids that support cardiac contractility and tissue perfusion. In this model, ITIH5 disrupts a necessary state within adipose that normally sustains vascular resistance and cardiac function.

In sum we leverage genetic network interactions to pinpoint the gene Itih5 as a driver of adipose tissue homeostasis. Acute and chronic inducible experimental systems in mice and human cells show that the protein plays a dual role in altering adipocyte differentiation and endothelial cell recruitment of immune cells to impact systemic metabolism and cardiac function. This new mechanism of adipocyte-derived regulation of immune cell recruitment presents a new mechanism to evaluate adipose tissue cellular homeostasis in cardiometabolic functions and disease.

## 4 Materials and methods

### 4.1 Mouse genetic network construction

All code and data to construct genetic networks are provided at https://github.com/mingqizh/HMDP-network-construction. Briefly, multi-tissue gene expression data from the hybrid mouse diversity panel [38] was used to run WGCNA to collapse genes into modules. Specifically, a matrix of strain (where genetic replicates imputed by strain average) ∼ gene_tissue expression was used as the input. Strains were then filtered for outlier detection using hierarchical clustering and blockwiseModules() were refined to produce an interpretable number of modules and observe membership within as well as across tissues. In the end, parameters used were power = 2, maxBlockSize = 5000, minModuleSize = 125, reassignThreshold = 1e-3 and mergeCutHeight = 0.42). Module eigengenes were then used to construct a network using igraph. Edges in the network were calculated based on the -log10(pvalue) of correlation significance (students pvalue based on bicor) between individual gene correlation with module eigengenes (Figure 1B). Cumulative module connectivity and significance were obtained based on the sum of -log10(regression pvalue) between for each module eigengenes across the whole network (Figure 1A). As noted above, central module drivers of ME4 were identified by corresponding to proteins which are known to be secreted (Uniprot definition of subcellular localization as “secreted”) and expression of a gene showing a significance (students pvalue of regression <1e-3) of, at least 2 traits shown in the network.

### 4.2 METSIM gene correlation and regression modeling

Expression and trait data from the Metabolic Syndrome in Man (METSIM) cohort were used to identify ITIH5 correlations with clinical traits and for regression-based mediation analyses. Gene ∼ trait correlations were calculated using the biweight micorrelation coefficient and corresponding students regression pvalue in WGCNA [39]. Regression mediation analyses were performed as previously described [42], correlations between traits or baseline and followup measures of the same traits were first calculated using WGCNA bicor. Next, residuals were calculated based on the correlation between ITIH5 and corresponding exposure (ex. baseline measure of trait), where comparisons made between either two traits (normal correlation) or ITIH5 ∼ trait residuals determined the level of ITIH5 covariation between the relationships.

### 4.3 Animal housing and experiments

All animal experiments have been approved by the UCI IACUC under protocol AUP-22-102. Specifically, these protocols have been designed to minimize the usage of animal experimentation to experiments which cannot be addressed through other models, as well as minimize the number of animals used in order to sufficiently test hypotheses proposed. Mice were maintained on a 12:12 light:dark cycle and monitored for changes in room temperature and humidity. Animals were euthanized using isoflurane followed by decapitation in order to collect sufficient amounts of blood and sera for downstream analyses. When animals were culled, tissues were snap-frozen in liquid nitrogen or placed in 4% formalin for histology.

### 4.4 Adeno-associated virus experiments

AAV creation was performed on a fee-for-service basis from Vectorbiolabs. Briefly, the mouse open reading frame for Itih5 (Gene ID: 209378) with an upstream human adiponectin promoter was PCR-amplified from and cloned into an expression system to yield AAV8 viral vectors containing the plasmid. AAV serotype 8 (AAV8) particles were packaged and purified where GFP-expressing vector (under the same promoter) was used as control. AAV8 particles were intraperitoneally injected at a dose of 2x10^12^ gc per mouse in 8–10 w old male C57BL/6N mice. After injection, the mice were assigned to dietary regiments (either normal show or HFD). AAV overexpression was quantified using qPCR. For the Chow diet cohort, samples sizes consisted of 5 AAV-GFP and 5 AAV-ITIH5 mice. The HFD cohort consisted of 7 AAV-GFP and 6 AAV-ITIH5 mice.

### 4.5 Recombinant protein injection

Recombinant human ITIH5 protein purified from human HEK293 cells was purchased from Origene (SKU TP314536L) and used for downstream experiments. For acute signaling experiments (Fig. 2), mice were fasted for 6 h then injected (IP) with 0.1ug protein/g body weight, as this dosage has been robust at seeing effects of soluble proteins [19]. For recombinant protein injections, a 6 h fasting time was selected as it has been standardized for normalizing whole-body metabolism in the context of assays such as glucose tolerance assays [77]. This timing is designed to ensure that circulating insulin levels have returned to baseline and blood glucose has stabilized without triggering significant compensatory gluconeogenesis or extreme weight loss [78]. N = 4C57BL/6N mice per group.

### 4.6 Bulk RNA-Seq

For bulk RNA-sequencing analysis, total RNA was extracted using chloroform/trizol fractionation, then supernatants containing RNA were placed in a Qiagen RNAmini column for binding, washing and purification according to manufacturer protocol. Total RNA was then shipped to Novogene for library preparation and sequencing. RNA was extracted using polyA beads and library preparation performed according to NEBNext RNA library kits. Libraries were sequenced on a Novo-Seq 6000 vie PE 150bp reads, targeting a read depth of 20million reads per sample. Fastq files were inspected for quality using fastQC then aligned to the mouse genome (GRCm38) using STAR. PCR duplicates were then removed from resulting BAM files using Markduplicates (Picard) and counts assembled at the gene-level using the summarizeOverlaps() function in GenomicAlignments. Differential expression based on injection was performed using DESeq2 and filtering genes which were detected (count >0) in over half the Veh and ITIH5 samples.

### 4.7 GTEx tissue-selective ITIH5 coregulation

Tissue-specific genetic correlation analyses can be performed and accessed via web tool (gdcat.org) or git repository (https://github.com/mingqizh/GD-CAT). Briefly, tissue data was accessed through GTEx V8 [79] and related analyses previously described [40]. Briefly, these data were filtered to retain genes which were detected across tissues where individuals were required to show counts >0 across all data. To quantify correlations within vs across tissues, biweight midcorrelation coefficients and corresponding p-values within and across tissues were generated using WGCNA bicorandpvalue() function [39]. Associated qvalue adjustments were applied using the Benjamini-Hochberg FDR from the R package “stats”. The BH procedure was selected instead of other FDR control methods because of its efficiency in CPU usage on the hosted server. Pathway enrichments were generated using gene set enrichment analyses available from the R package clusterProfiler. Specifically, the bicor coefficients were used as the rank-weight of each gene and enrichment tests performed by permuting against the human or mouse reference transcriptome. Terms used for the enrichment analyses were derived from Gene Ontology (Biological Process, Cellular Component and Molecular Function) which were accessed using the R package enrichR.

### 4.8 Adipocyte stem cell isolation

After CO2 asphyxiation followed by cervical dislocation gonadal adipose tissue (gWAT) was harvested from 10-week-old male C57bl/6 mice on a normal chow diet. Mice were placed on a surgical bench protector inside a laminar flow hood where fur was saturated with 70% EtOH prior to surgical procedure. Harvested fat pads were first placed in a 10 cm dish and washed with HBSS (Gibco) then transferred to the corresponding dish lid where the pads were minced using sterile surgical scissors. Minced tissue was transferred to a sterile 50 ml centrifuge tube containing Type I collagenase (Sigma–Aldrich), 3.3U DNase 1 (Roche) and 1 mM MgCl (Invitrogen) then placed in a 37˙C shaker for 1 h at 100rpm. Once digested, the tissue was run through a sterile 100 μm filter (GenClone) and centrifuged at 500×*g* for 5 min at RT. Post centrifugation the supernatant was aspirated and replaced with 2 ml of ACK red cell lysis buffer (Gibco) followed by gentle resuspension via trituration and left to incubate for 4 min. The ACK was neutralized by the addition of 10 ml of HBSS. The diluted cell mixture was then centrifuged at 500×*g* for 5 min. Following centrifugation the supernatant was removed and replaced with ASC harvest media – DMEM/F12 (Gibco), 15% FBS (GenClone), 1x antibiotic solution of Penicillin/Streptomycin (Corning) – resuspended with gentle trituration and evenly distributed into a 6 well plate (GenClone). ASC harvest media was replaced with fresh harvest media 24 h after plating. Thereafter the media was changed every 2–3 days until cells reached ∼90% confluency at which time the ASC harvest media was removed and replaced with ASC differentiation media – Harvest media spiked with 33 μM biotin (Sigma–Aldrich), 17 μM d-pantothenate (Sigma–Aldrich), 1 μM dexamethasone (Sigma–Aldrich), 5 μM rosiglitazone (Sigma–Aldrich), 500 μM methylisobutylxanthine (Sigma–Aldrich), 100 nM insulin (Sigma–Aldrich) – and left for 3 days in one of four groups: control, 100 ng/mL + itih5 (OriGene), 5 μM + IWP-2 Wnt inhibitor (MedChemExpress) or + itih5 and IWP-2 Wnt inhibitor. On day four post differentiation the ASC differentiation media was removed and replaced with ASC propagation media: Differentiation media without the addition of methylisobutylxanthine and rosiglitazone, but continuing the +/− itih5 and/or +/− IWP-2. The cells were kept in ASC propagation media for approximately 12 days post-induction changing the media every 2–3 days.

### 4.9 Oil Red O staining

Cells were washed with PBS which was later removed, replaced with 10% formalin (Epredia) and incubated for 60 min at RT. Formalin was removed, cells were washed 2x with ddH2O and then incubated in 60% 2-propanol for 5 min at RT. We note that the use of 60% isopropanol has been standardized as part of Oil Red O staining in adipocytes [80]. The 60% 2-propanol was removed completely, replaced with Oil Red O solution (Sigma–Aldrich) and left to incubate for 10 min. Immediately following the incubation period the Oil Red O solution was removed, the cells were washed 4x with ddH2O and images were acquired (Leica DMi8).

### 4.10 Endothelial single-cell atlas integration

Single-cell RNA sequencing data from 19 human organs and tissues, comprising approximately 67,000 vascular cells from 62 donors, were obtained from the Human Vascular Cell Atlas [52]. A total of 42 distinct vascular cell states were identified. DEGs associated with specific cell types and states were determined using the Wilcoxon Rank Sum test (p > 0.05). To perform cross-species integration, human DEGs were intersected with DEGs identified from the recombinant ITIH5 injection mouse model. Orthologous gene mappings between human and mouse were retrieved using Ensembl BioMart to ensure consistent gene annotation and facilitate comparative analysis.

### 4.11 Adhesion assay

This assay was performed as previously described [53,81]. HAECs were stimulated for adhesion for 5 h in RPMI media (Corning Scientific) containing 5% FBS (Atlanta Biologicals) with indicated treatments. At 2 h prior to assay, leukocytes were spun down at 300*g* for 10 min and pelleted. Cells were then resuspended in PBS containing 25 μmol/L Vybrant CFDA SE Cell Tracer (Life Technologies) for 15 min at 37 °C. Labeled leukocytes were then spun down at 1000 rpm for 10 min and resuspended in RPMI media (Corning Scientific) with 5% FBS (Atlanta Biologicals). The spin and resuspension were repeated twice more to remove excess dye. Leukocytes were then added to HAECs for 30 min to allow adhesion. The combined cell mixture was then washed 3 × with PBS to remove unbound leukocytes. Finally, cells were fixed in 4% paraformaldehyde and imaged. Quantification for adhesion assays were performed by placing a 1-mm grid over each image and counting the number of fluorescent points per 100-μm square. Each mean value reflects 9 images taken, 3 each from separate donors.

### 4.12 Adipose tissue histology

GWAT tissues were fixed overnight in 10% Formalin, embedded in paraffin, and processed onto slides for downstream hematoxylin-eosin (H&E) as well as specific antibody probing. GWAT fibrosis was assessed using Sirius Red, whereby adipose sections were rehydrated in descending ethanol concentrations and then incubated for 2 h at room temperature with an aqueous solution of saturated picric acid containing 0.1% Fast Green FCF and 0.1% Direct Red. Primary monoclonal antibodies used to perform immunostaining were: anti-F4/80 (a murine pan-macrophage marker; 1:50) (AbDSerotec, Hercules, CA, USA), anti-alpha smooth muscle actin (aSMA; 1:500) (Abcam, Waltham, MA, USA) and anti-neutrophil elastase (1:1200) (Abcam, Waltham, MA, USA). Negative controls have primary antibodies omitted. The adipose specimens were deparaffinized and slowly rehydrated using descending ethanol concentrations. The antigens were retrieved in citrate buffer pH 6.0 for 30 min at 95 °C, or treated with 2% BSA 1x Triton in TBS-T for 30 min at room temperature. Following overnight incubation with primary antibodies, horseradish peroxidase (HRP) secondary antibody was applied. For color reaction of HRP, a solution of 3, 3-diaminobenzidine (DAB) was used as chromogen and the slides were counterstained with hematoxylin. All photos were taken at 10× magnification using the NanoZoomer 2.0HT Slide Scanning System (Hamamatsu, Japan). A total of five fields per sample were examined. The stained-area per field of aSMA as well as F4/80 was determined using ImageJ software (National Institute of Health, USA). Briefly, color images were separated into RGB channels, and the green channel was removed. The resulting images were converted to 8-bit grayscale, and a user-defined threshold was applied to measure the positive staining area and area fraction (percentage).

### 4.13 Spatial RNA-Seq analysis

Adipose tissue from mice subjected to a 12-week HFD was extracted and processed using the Visium Spatial for FFPE Gene Expression Kit (Mouse Transcriptome, 4 reactions). Samples were prepared on Visium Tissue Section Test Slides with the Visium CytAssist (10x Genomics). The Visium platform was facilitated by the Genomics Research and Technology Hub at the University of California, Irvine. Initial data processing was performed using the Voyager package, which included quality control, normalization, and retention of reads within tissue boundaries, followed by Principal Component Analysis (PCA). Cell segmentation performed on H&E images from Visium: First, contrast was increased. Then simple thresholding was performed to segment the adipocyte fatty droplets with the EBImage R package. Finally the segmentation mask was manually corrected with QuPath to account for broken adipocytes, and the mask was converted into polygons with the sf and terra R packages. The Visium data allowed for the calculation of autocorrelation based on spatial gene expression levels, providing insights into the sparsity or clustering of gene expression. Using runBivariate() to perform the Local Lee's analysis, the spatial co-expression of genes within single barcodes was evaluated, focusing on immune markers, adipocyte cell markers, endothelial markers, and immune recruitment markers to investigate whether ITIH5 suppresses immune pathways via endothelial cells. Statistical significance was assessed using p-values adjusted by the Benjamini & Hochberg method to control for false discovery rates in multiple testing, ensuring robust and reliable results. Single-cell algorithms were integrated into the Visium spatial data. SCTransform and Harmony integration were performed before cell type clustering at a resolution of 0.5, leading to the identification of 11 distinct cell types. These cell types were remapped to spatial slices using conserved genes as markers via FindConservedMarkers() function. Differential expression analysis was conducted to compare ITIH5 overexpression and control groups within each cluster. For this, the FindClusters() function was utilized, specifying experimental groups based on the combination of genotype and cluster. DE genes were identified using the MAST test, which is robust for single-cell and sparse data. The criteria for inclusion included a minimum detection fraction of 25% and a log-fold change threshold of 0.25. Subsequently, gene set enrichment analysis (GSEA) was performed on the dendritic cell and B cell DEGs to identify enriched biological pathways and functional processes associated with ITIH5 treatment. This analysis provided insights into the molecular mechanisms underlying the observed transcriptional changes in immune cell populations.

### 4.14 Echocardiography

After anesthesia induction with isoflurane, animals were maintained under inhaled 1.5% isoflurane and 95% O_2_ to allow for spontaneous breathing. Animals were imaged in a left lateral decubitus position and warming pads were used to maintain normothermia. Images of horizontal long axis view and short axis view of the heart were collected in both M-mode and B-mode using a Vevo 3100 ultrasound system with 25-mHz scan head (VisualSonics, Toronto, ON, Canada). The “LV trace” feature was used to calculate LV structural and functional parameters from the LV parasternal short-axis M-mode view, which was recorded at the level of two papillary muscles. LV posterior wall thickness during diastole was measured on B-mode long axis view. Vevo analysis software (VevoLab, VisualSonics) was used to conduct all measurements and calculations. Vevo software analysis was performed by a research team member blinded to study groups.

### 4.15 Glucose tolerance tests

Glucose tolerance tests were performed as previously described [19,82]. Mice were fasted for 8 h where baseline glucose measurements were taken, then injected with 10% wt:vol of a glucose:saline solution. All glucose measurements were taken from the tail using a glucometer (Accucheck plus).

### 4.16 Plasma hormone assays

Plasma metabolic hormones, including insulin, C-peptide 2, GLP-1, IL-6, leptin, CCL2, TNF-alpha, and resistin, were measured using the Luminex-based multiplex Mouse Metabolic Hormone Expanded Panel assay kit (MilliporeSigma, Cat. #MMHE-44K) according to manufacturers specifications and analyzed on a Lumix plate-reader.

## CRediT authorship contribution statement

**Mingqi Zhou:** Methodology, Investigation, Formal analysis, Data curation, Conceptualization. **Marcus Seldin:** Writing – review & editing, Writing – original draft, Validation, Supervision, Project administration, Funding acquisition, Formal analysis, Conceptualization.

## Funding support

This work was supported by NIH grants R00-HL138193, DP1-DK130640 and U54-OD039864. This work utilized resources of the UCI Genomics Research and Technology Hub (GRT Hub) parts of which are supported by NIH grants to the Comprehensive Cancer Center (P30CA-062203) and the UCI Skin Biology Resource Based Center (P30AR075047) at the University of California, Irvine, as well as to the GRT Hub for instrumentation (1S10OD010794-01and 1S10OD021718-01).

## Declaration of competing interest

The authors confirm that there are no competing interests to declare.

## Data Availability

Data will be made available on request.

## Appendix A Supplementary data

Figure S1 **A-E**, In the METSIM cohort, baseline and 5-year follow-up BMI were strongly correlated among the 774 patients where RNA-seq was performed (**A**). Expression of *ITIH5* in adipose tissue was significantly positively correlated with both baseline (**B**) and follow-up BMI (**C**). To see if the pattern of variance between *ITIH5* and baseline BMI could explain the strong correlation between baseline and follow-up BMI, we repeated the regression between baseline and following BMI while adding *ITIH5* expression as a covariate to the model (ex. regressing out), where a strong drop in significance was observed (**D**). **E**, These analyses are summarized by plotting the -log10(regression Pvalue) between clinical traits, *ITIH5* and adjusted regressions. **F-J**. The same analyses shown in **A-E** in comparing baseline to follow-up measures with respect to *ITIH5* variation using different clinical traits. **K–O**, The same analyses as E-J are shown but instead of comparing baseline to follow-up measures for the same clinical trait, are analyzed for correlation between clinical traits.

Figure S10 Non-reducing gels showing bikunin protein levels in adipose tissue and serum. Because ITIH family members have been shown to form complexes with bikunin, immunoblotting was performed on serum and adipose tissue from mice overexpressing either AAV-GFP or AAV-ITIH5. A, Blotting for bikunin shows two distinct bands, a lower molecular weight band at 130 kDa showing bikunin bound to ITIH and a higher molecular weight (>200 kDa) showing higher order bikunin self-complexing (unbound to ITIH). Quantification of the bound complex at 130 kDa was used as a proxy for the amount of ITIH-bikunin complex, where no significant differences were observed between AAV groups in either adipose tissue (B) or Serum (C).

Figure S11 Violin plots displaying the normalized expression (logcounts) of immunoglobulin genes of Igkc (A) and Ighg2b (B). (C) B cell GSEA network highlighting the suppression of core functions, including antibody production and fatty acid metabolism. Node size indicates gene count; color indicates enrichment p-value. (D) Spatial transcriptomics mapping utilizing Local Lee's bivariate statistic to evaluate the spatial co-clustering of Itih5 and the antibody secretion gene Ighj4.

Figure S12 Spatial co-expression analysis Local Lee's using runBivariate() reveals barcode-level gene correlations across endothelial, adipocyte, and immune markers under ITIH5 treatment. Under ITIH5 treatment, endothelial-mediated immune cell recruitment is markedly suppressed.

Figure S13 Plasma levels of C-Peptide, GLP-1, IL-6, CCL2, and TNF-alpha were measured via Luminex multiplex assay (MilliporeSigma #MMHE-44K). Graphs compare GFP control and ITIH5-overexpressing mice on either standard chow or HFD. Statistical significance was determined by Student's t-test.

Multimedia component 15

Figure S2 Adipose tissue expression of Itih5 (y-axis) in matched strains from the hybrid mouse diversity panel compared between normal chow or HF/HS feeding regiments (x-axis). Lines indicated paired strains, pvalue based on wilcoxon t-test.

Figure S3 Normalized *ITIH5* expression (y-axis) from cell types produced (x-axis) during adipose tissue fractionation[49].

Figure S4 Schematic design and RNA-seq similar to experiments shown in Figure 2; however, mice were fed a HF/HS diet for 8 weeks prior to injection of recombinant protein.

Figure S5 Oil Red O staining of adipocytes differentiated from ASC cultures derived from gWAT of 10-week-old male C57bl/6 mice. Cells were treated for 3 days with control media, 100 ng/mL Itih5, 5 μM IWP-2 Wnt inhibitor, or both itih5 and IWP-2. Lipid accumulation was visualized after 12 days post-induction, revealing treatment-specific differences in adipocyte morphology and lipid content.

Figure S6 Dot plot visualization of activated and suppressed pathways from cross-species comparison between human vascular single-cell RNA sequencing data and the ITIH5 mouse model.

Figure S7 Body weight and fat mass measured over 12 weeks for mice expressing AAV-GFP (control, grey) or AAV-ITIH5 (orange). Experiments were conducted under standard chow diet conditions or a HF diet. Statistical significance between AAV-GFP and AAV-ITIH5 groups at each time point was determined using a Wilcoxon test, with p-values indicated above the respective data points. A statistically significant difference (p = 0.013, bold in red) was observed in the body weight of mice on the HF diet at week 5.

Figure S8 Percentage of positive stained area for aSMA, F4/80, and Sirius Red. Histological sections were evaluated from mice expressing either AAV-GFP or AAV-ITIH5, maintained on normal chow or a HF/HS diet. Image analysis and quantification were performed using ImageJ software.

Figure S9 Histological and immunoblot comparisons of Liver (top) and muscle (bottom) between AAV-GFP and AAV-ITIH5 mice on normal chow and HF/HS diets. No notable differences were observed between groups.
