# Supplementary material for: Systems genetics reveals ITIH5 as a key mediator of adipocyte–Endothelial crosstalk
Source: Mol Metab. 2026 Apr 21;108:102373. doi: 10.1016/j.molmet.2026.102373 (PMC13156600; doi:10.1016/j.molmet.2026.102373)
Supplement: Figure S1 — A-E, In the METSIM cohort, baseline and 5-year follow-up BMI were strongly correlated among the 774 patients where RNA-seq was performed (A). Expression of ITIH5 in adipose tissue was significantly positively correlated with both baseline (B) and follow-up BMI (C). To see if the pattern of variance between ITIH5 and baseline BMI could explain the strong correlation between baseline and follow-up BMI, we repeated the regression between baseline and following BMI while adding ITIH5 expression as a covariate to the model (ex. regressing out), where a strong drop in significance was observed (D). E, These analyses are summarized by plotting the -log10(regression Pvalue) between clinical traits, ITIH5 and adjusted regressions. F-J. The same analyses shown in A-E in comparing baseline to follow-up measures with respect to ITIH5 variation using different clinical traits. K–O, The same analyses as E-J are shown but instead of comparing baseline to follow-up measures for the same clinical trait, are analyzed for correlation between clinical traits. [file mmc1.pdf]

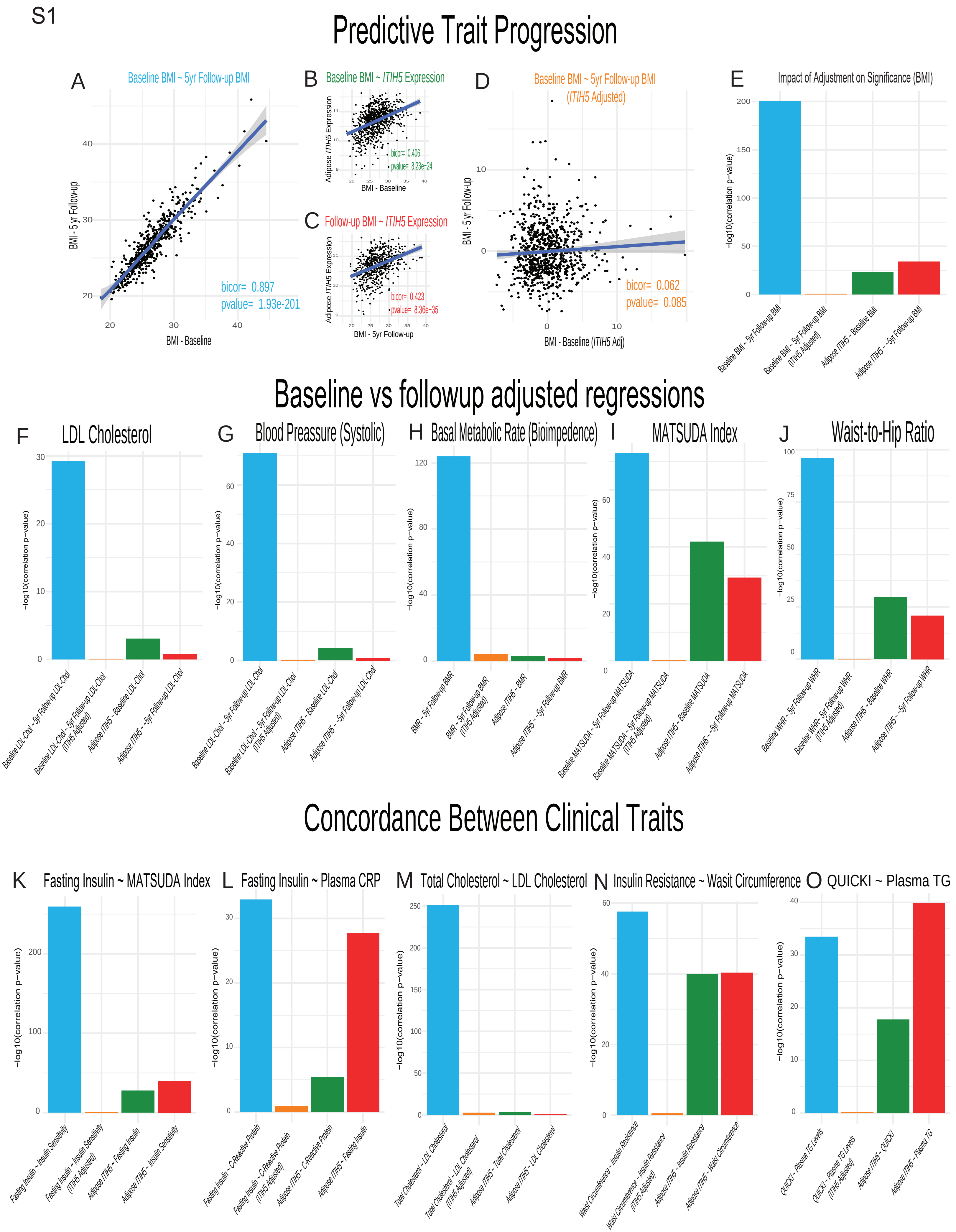
S1
Predictive Trait Progression
A
Baseline BMI ~ 5yr Follow-up BMI
BMI - 5 yr Follow-up
BMI - Baseline
bicor= 0.897
pvalue= 1.93e-201
B
Baseline BMI ~ ITIH5 Expression
Adipose ITIH5 Expression
BMI - Baseline
bicor= 0.406
pvalue= 8.23e-24
C
Follow-up BMI ~ ITIH5 Expression
Adipose ITIH5 Expression
BMI - 5yr Follow-up
bicor= 0.423
pvalue= 8.36e-35
D
Baseline BMI ~ 5yr Follow-up BMI
(ITIH5 Adjusted)
BMI - 5 yr Follow-up
BMI - Baseline (ITIH5 Adj)
bicor= 0.062
pvalue= 0.085
E
Impact of Adjustment on Significance (BMI)
−log10(correlation p−value)
Baseline BMI - 5yr Follow-up BMI
Baseline BMI - 5yr Follow-up BMI (ITIH5 Adjusted)
Adipose ITIH5 - Baseline BMI
Adipose ITIH5 - -5yr Follow-up BMI
Baseline vs followup adjusted regressions
F
LDL Cholesterol
G
Blood Preassure (Systolic)
H
Basal Metabolic Rate (Bioimpedence)
I
MATSUDA Index
J
Waist-to-Hip Ratio
−log10(correlation p−value)
Baseline LDL-Chol - 5yr Follow-up LDL-Chol
Baseline LDL-Chol - 5yr Follow-up LDL-Chol (ITIH5 Adjusted)
Adipose ITIH5 - Baseline LDL-Chol
Adipose ITIH5 - -5yr Follow-up LDL-Chol
BMR - 5yr Follow-up BMR
BMR - 5yr Follow-up BMR (ITIH5 Adjusted)
Adipose ITIH5 - BMR
Adipose ITIH5 - -5yr Follow-up BMR
Baseline MATSUDA - 5yr Follow-up MATSUDA
Baseline MATSUDA - 5yr Follow-up MATSUDA (ITIH5 Adjusted)
Adipose ITIH5 - Baseline MATSUDA
Adipose ITIH5 - -5yr Follow-up MATSUDA
Baseline WHR - 5yr Follow-up WHR
Baseline WHR- 5yr Follow-up WHR (ITIH5 Adjusted)
Adipose ITIH5 - Baseline WHR
Adipose ITIH5 - -5yr Follow-up WHR
Concordance Between Clinical Traits
K
Fasting Insulin ~ MATSUDA Index
L
Fasting Insulin ~ Plasma CRP
M
Total Cholesterol ~ LDL Cholesterol
N
Insulin Resistance ~ Wasit Circumference
O
QUICKI ~ Plasma TG
−log10(correlation p−value)
Fasting Insulin - Insulin Sensitivity
Fasting Insulin - Insulin Sensitivity (ITIH5 Adjusted)
Adipose ITIH5 - Fasting Insulin
Adipose ITIH5 - Insulin Sensitivity
Fasting Insulin - C-Reactive Protein
Fasting Insulin - C-Reactive Protein (ITIH5 Adjusted)
Adipose ITIH5 - C-Reactive Protein
Adipose ITIH5 - Fasting Insulin
Total Cholesterol - LDL Cholesterol
Total Cholesterol - LDL Cholesterol (ITIH5 Adjusted)
Adipose ITIH5 - Total Cholesterol
Adipose ITIH5 - LDL Cholesterol
Waist Circumference - Insulin Resistance
Waist Circumference - Insulin Resistance (ITIH5 Adjusted)
Adipose ITIH5 - Insulin Resistance
Adipose ITIH5 - Waist Circumference
QUICKI - Plasma TG Levels
QUICKI - Plasma TG Levels (ITIH5 Adjusted)
Adipose ITIH5 - QUICKI
Adipose ITIH5 - Plasma TG
