# Supplementary figures and images for: Systems genetics reveals ITIH5 as a key mediator of adipocyte–Endothelial crosstalk
Source: Mol Metab. 2026 Apr 21;108:102373. doi: 10.1016/j.molmet.2026.102373 (PMC13156600; doi:10.1016/j.molmet.2026.102373)

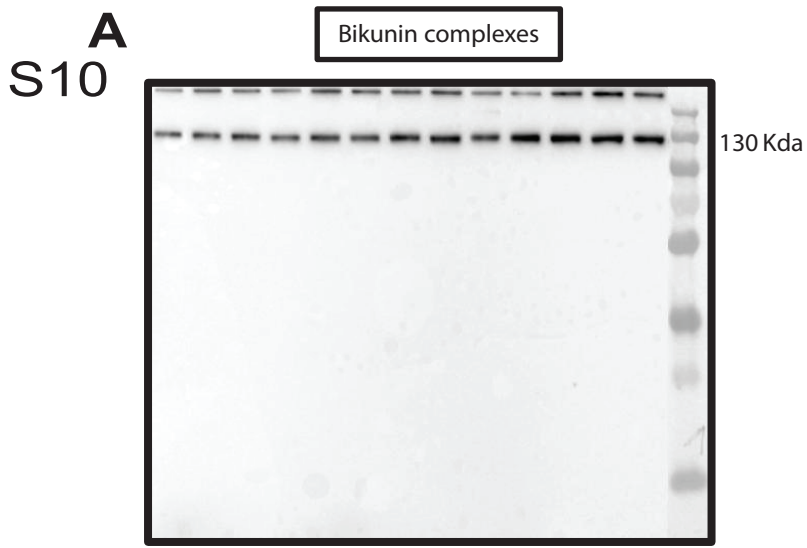

**B** Bikunin-HC(?) (130 Kda) protein expression adipose

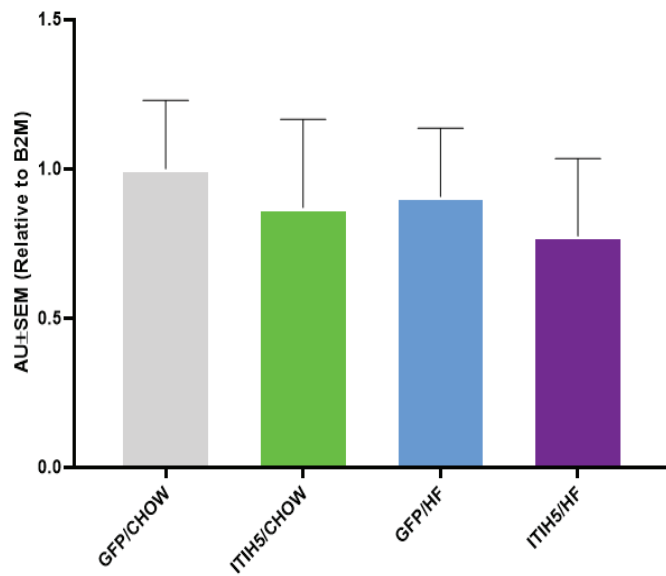

**C** Serum Bikunin-CS-HC(?) (130 Kda) protein levels

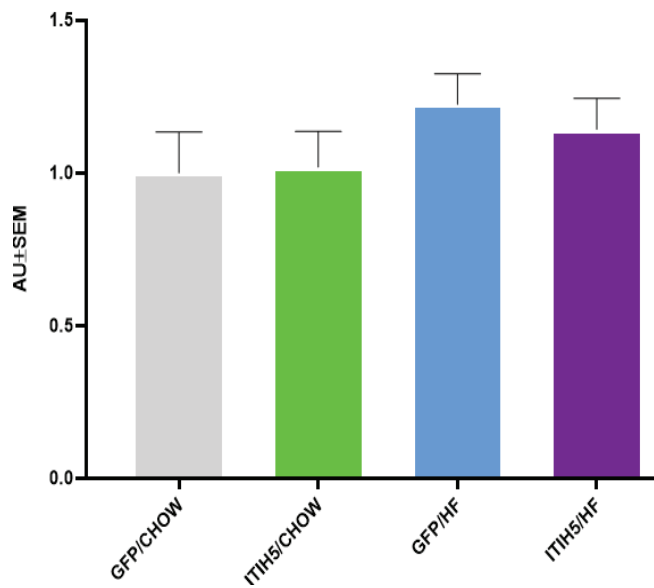

Supplement: Figure S10 — Non-reducing gels showing bikunin protein levels in adipose tissue and serum. Because ITIH family members have been shown to form complexes with bikunin, immunoblotting was performed on serum and adipose tissue from mice overexpressing either AAV-GFP or AAV-ITIH5. A, Blotting for bikunin shows two distinct bands, a lower molecular weight band at 130 kDa showing bikunin bound to ITIH and a higher molecular weight (>200 kDa) showing higher order bikunin self-complexing (unbound to ITIH). Quantification of the bound complex at 130 kDa was used as a proxy for the amount of ITIH-bikunin complex, where no significant differences were observed between AAV groups in either adipose tissue (B) or Serum (C). [file mmc10.pdf]

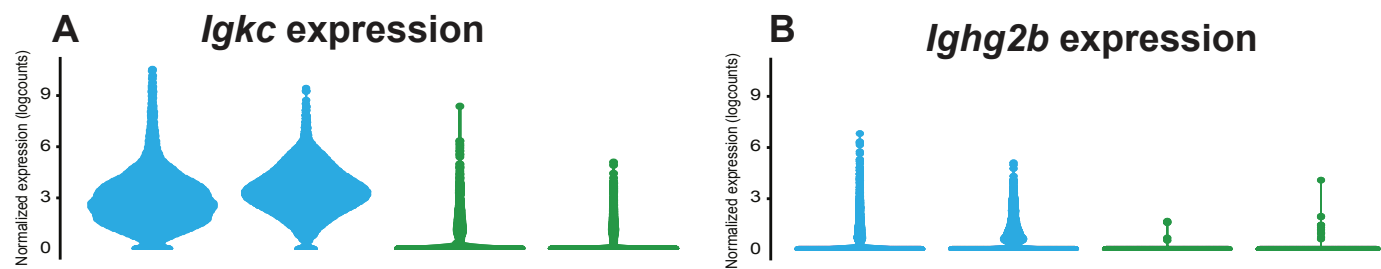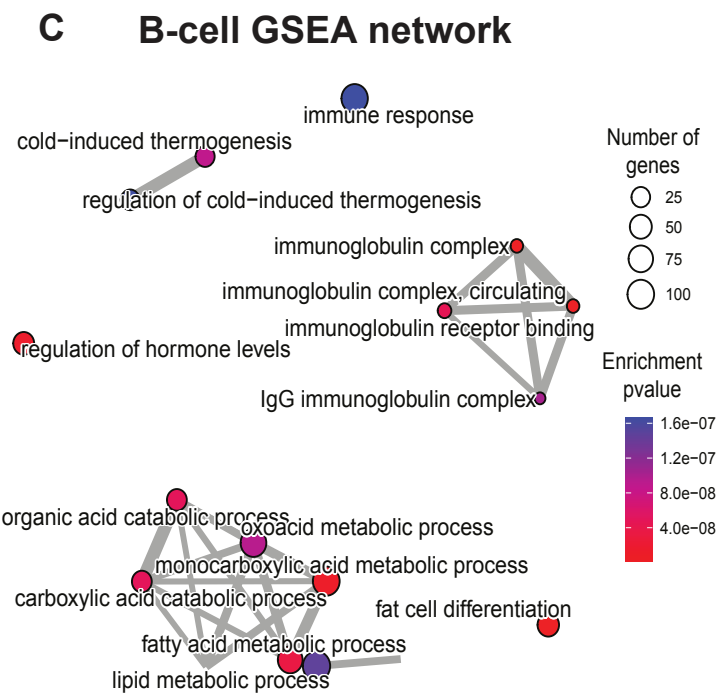

**D** Local Lee's bivariate statistic

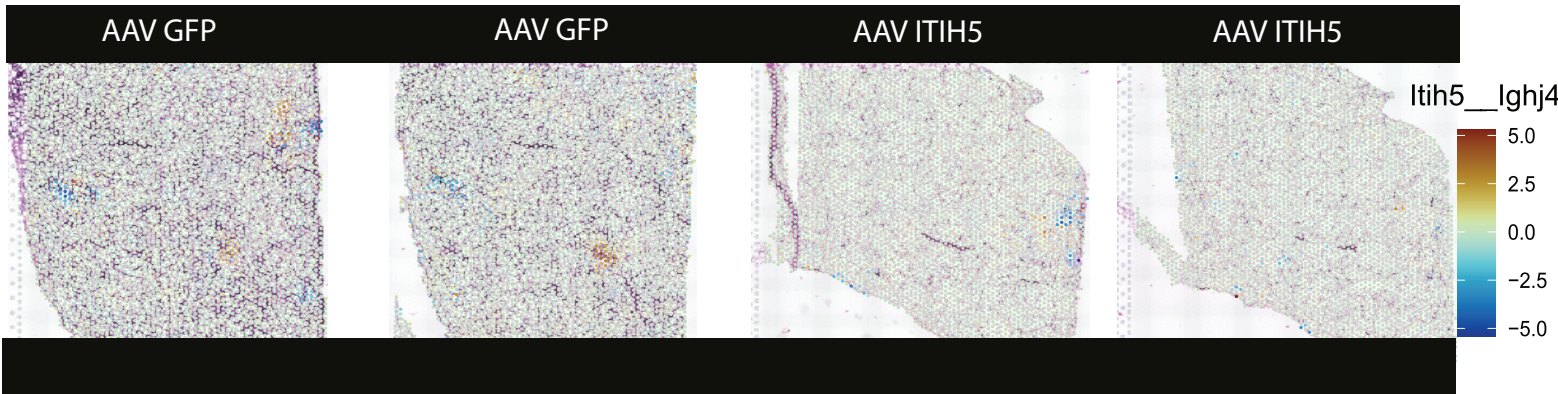

Supplement: Figure S11 — Violin plots displaying the normalized expression (logcounts) of immunoglobulin genes of Igkc (A) and Ighg2b (B). (C) B cell GSEA network highlighting the suppression of core functions, including antibody production and fatty acid metabolism. Node size indicates gene count; color indicates enrichment p-value. (D) Spatial transcriptomics mapping utilizing Local Lee's bivariate statistic to evaluate the spatial co-clustering of Itih5 and the antibody secretion gene Ighj4. [file mmc11.pdf]

AAV-GFP

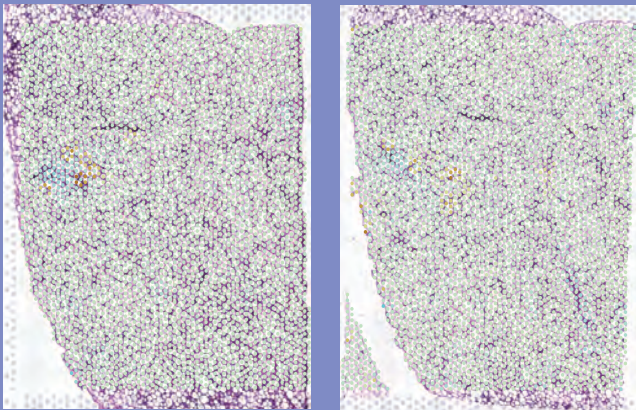

AAV-ITI5

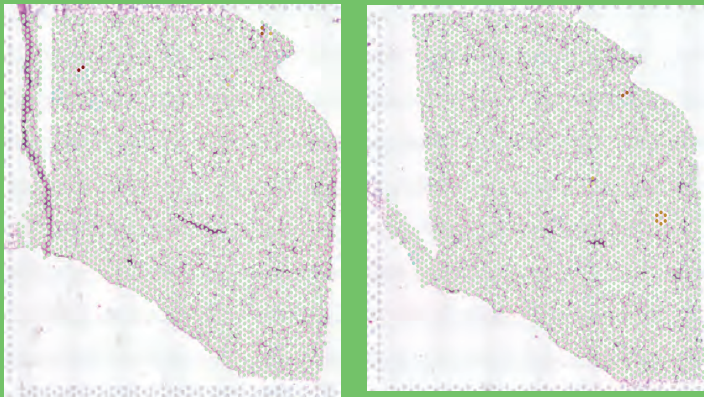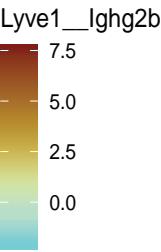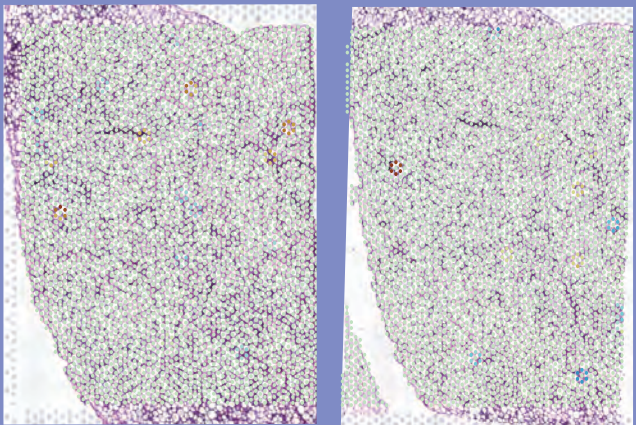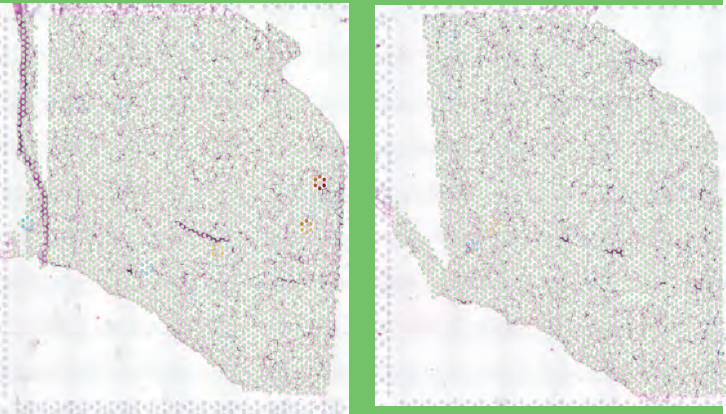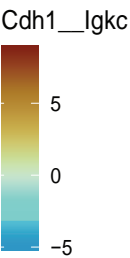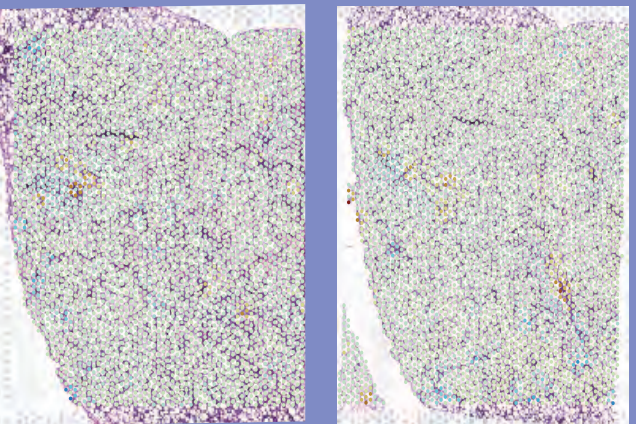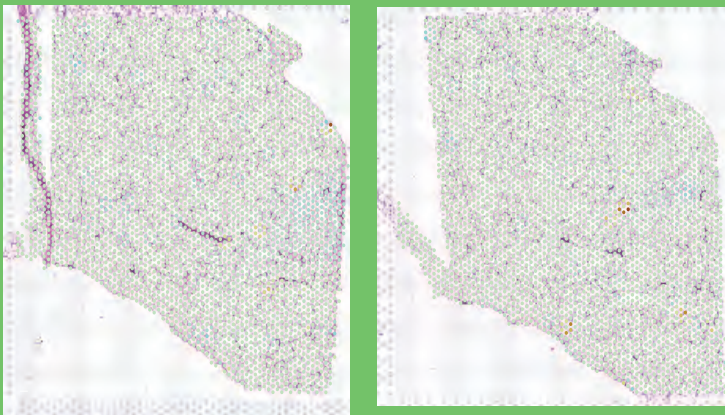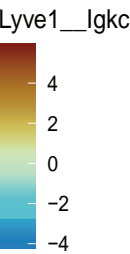

Supplement: Figure S12 — Spatial co-expression analysis Local Lee's using runBivariate() reveals barcode-level gene correlations across endothelial, adipocyte, and immune markers under ITIH5 treatment. Under ITIH5 treatment, endothelial-mediated immune cell recruitment is markedly suppressed. [file mmc12.pdf]

S13

group    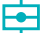 GFP\_Chow    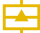 ITIH5\_Chow    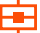 GFP\_HFD    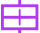 ITIH5\_HFD

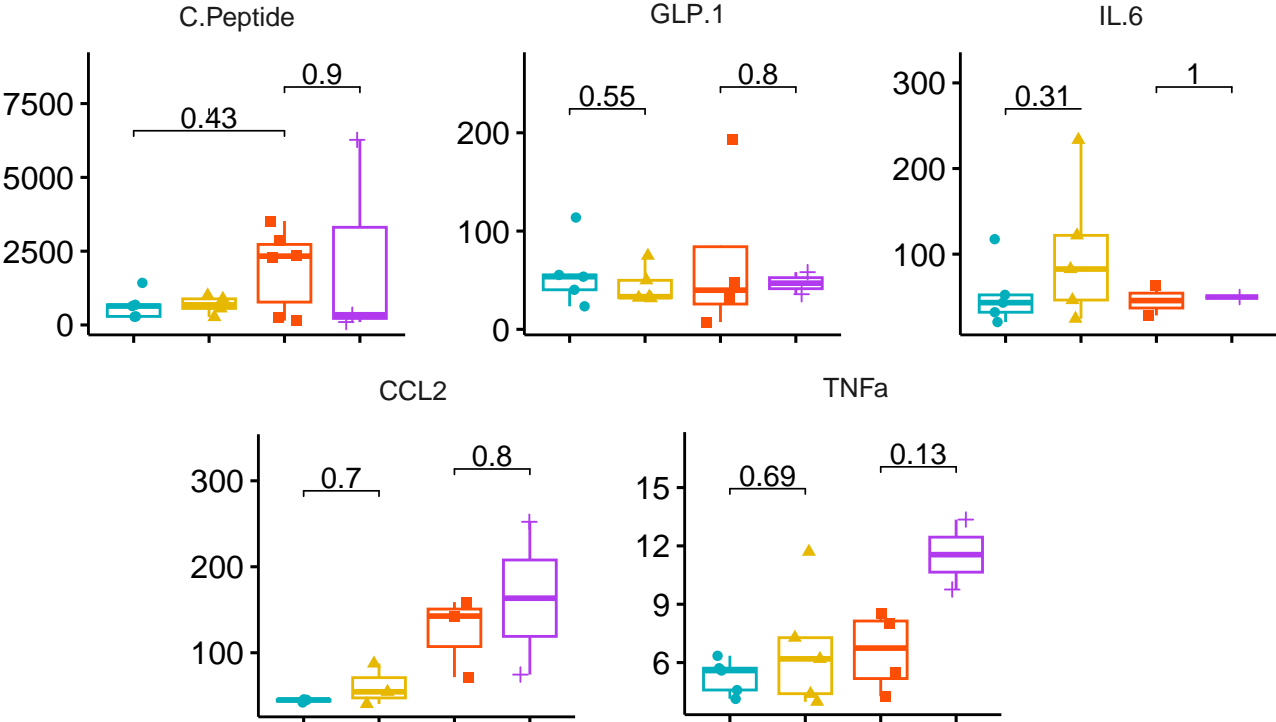

Supplement: Figure S13 — Plasma levels of C-Peptide, GLP-1, IL-6, CCL2, and TNF-alpha were measured via Luminex multiplex assay (MilliporeSigma #MMHE-44K). Graphs compare GFP control and ITIH5-overexpressing mice on either standard chow or HFD. Statistical significance was determined by Student's t-test. [file mmc13.pdf]

S2      Dietary effects on adipose Itih5 expression

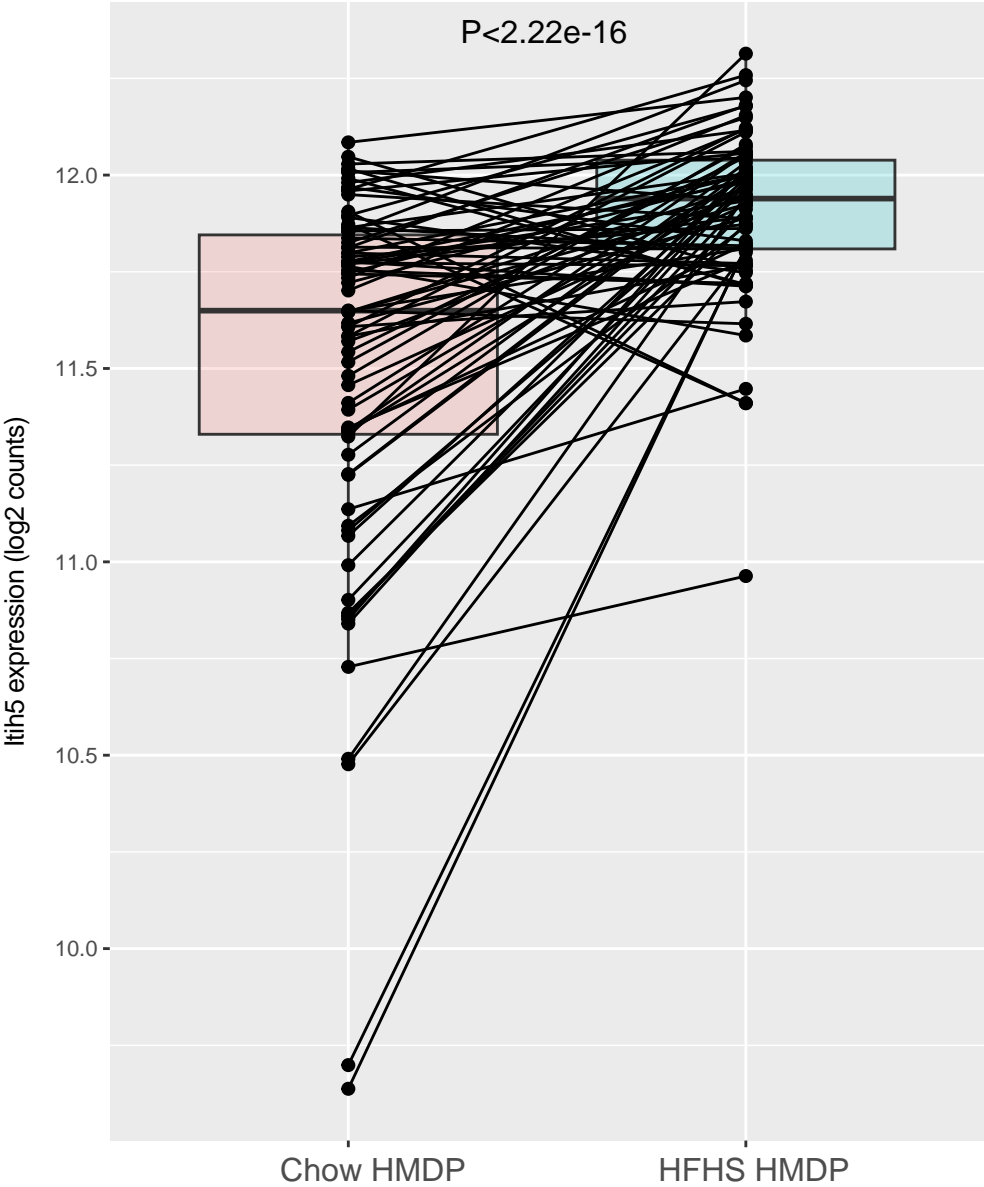

Supplement: Figure S2 — Adipose tissue expression of Itih5 (y-axis) in matched strains from the hybrid mouse diversity panel compared between normal chow or HF/HS feeding regiments (x-axis). Lines indicated paired strains, pvalue based on wilcoxon t-test. [file mmc2.pdf]

Human ITIH5 expression  
(adipose fractionation,  
80 females)

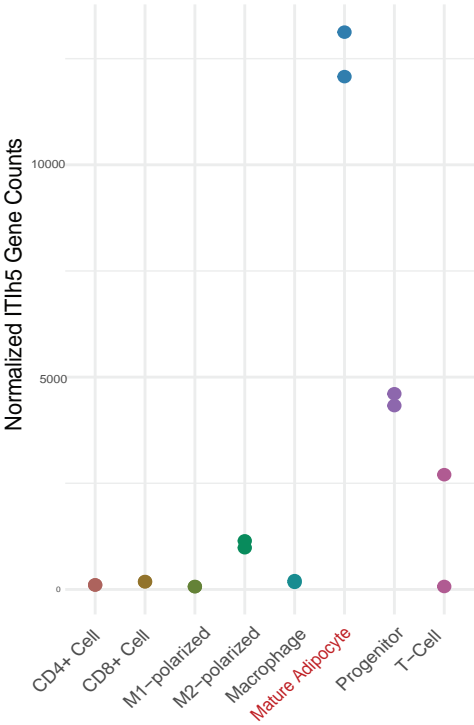

Supplement: Figure S3 — Normalized ITIH5 expression (y-axis) from cell types produced (x-axis) during adipose tissue fractionation[49]. [file mmc3.pdf]

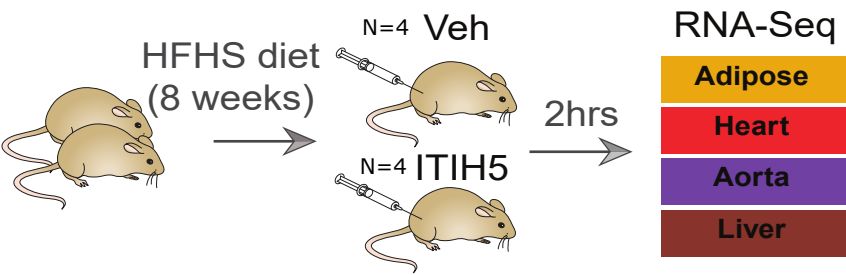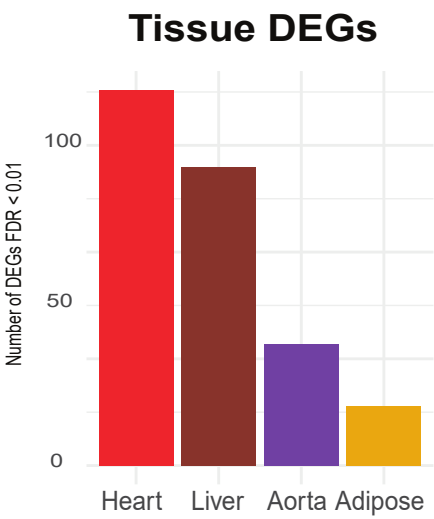

Supplement: Figure S4 — Schematic design and RNA-seq similar to experiments shown in Figure 2; however, mice were fed a HF/HS diet for 8 weeks prior to injection of recombinant protein. [file mmc4.pdf]

## S5 Mouse primary adipocyte stem cell differentiation (Oil red O)

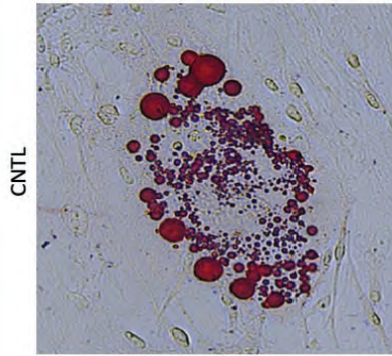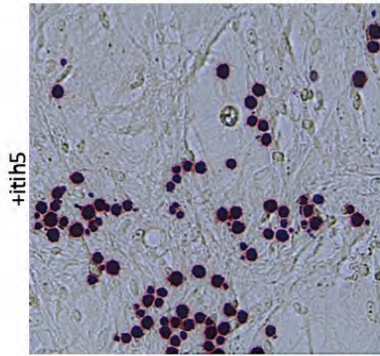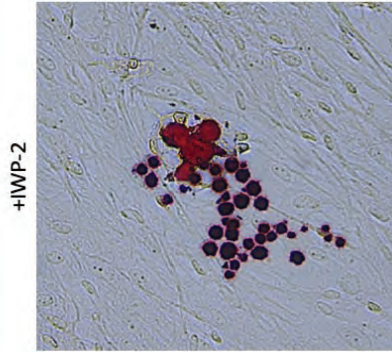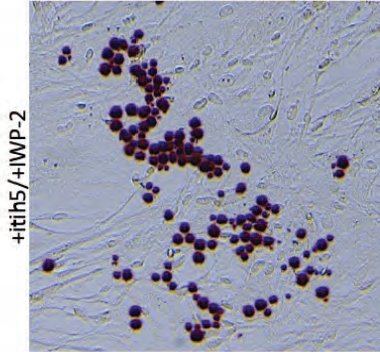

Supplement: Figure S5 — Oil Red O staining of adipocytes differentiated from ASC cultures derived from gWAT of 10-week-old male C57bl/6 mice. Cells were treated for 3 days with control media, 100 ng/mL Itih5, 5 μM IWP-2 Wnt inhibitor, or both itih5 and IWP-2. Lipid accumulation was visualized after 12 days post-induction, revealing treatment-specific differences in adipocyte morphology and lipid content. [file mmc5.pdf]

# Non-Endothelial pathways

S6

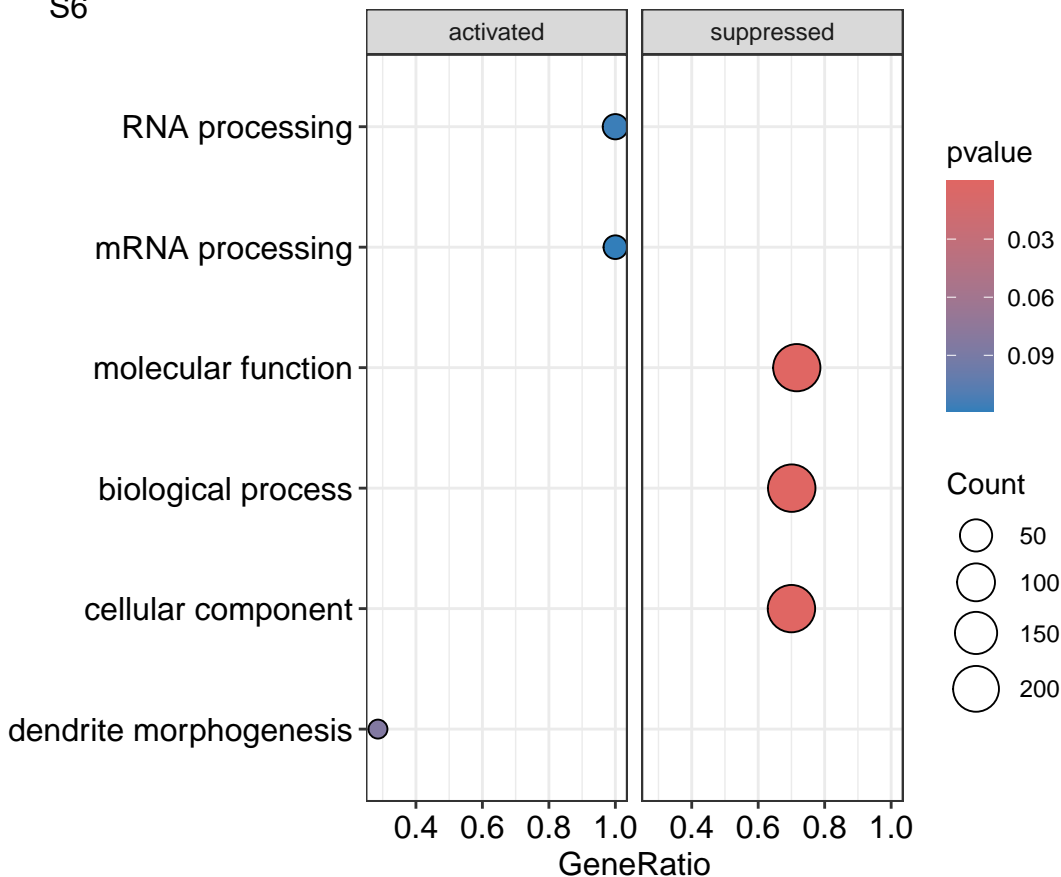

Supplement: Figure S6 — Dot plot visualization of activated and suppressed pathways from cross-species comparison between human vascular single-cell RNA sequencing data and the ITIH5 mouse model. [file mmc6.pdf]

Body weight - Chow diet

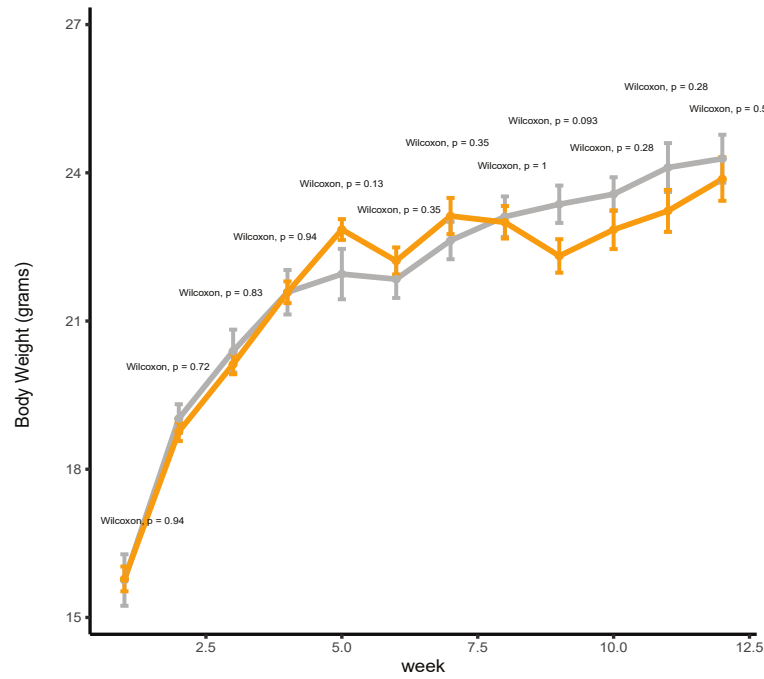

Fat mass - chow diet

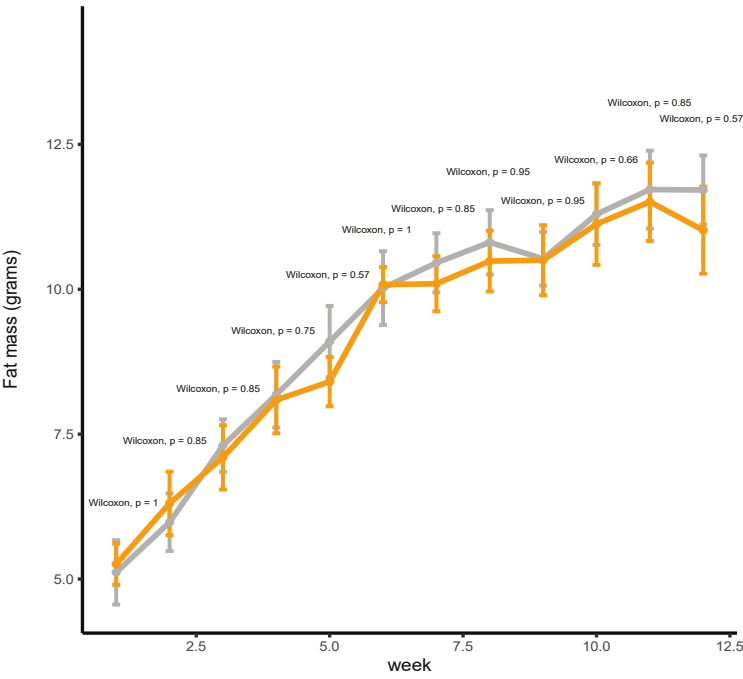

Body weight - HF diet

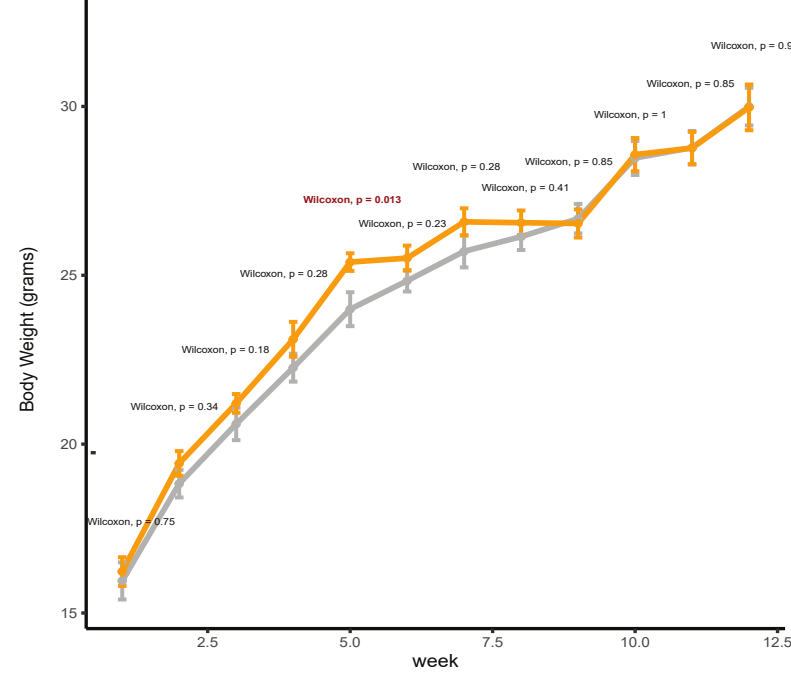

Fat mass - HF diet

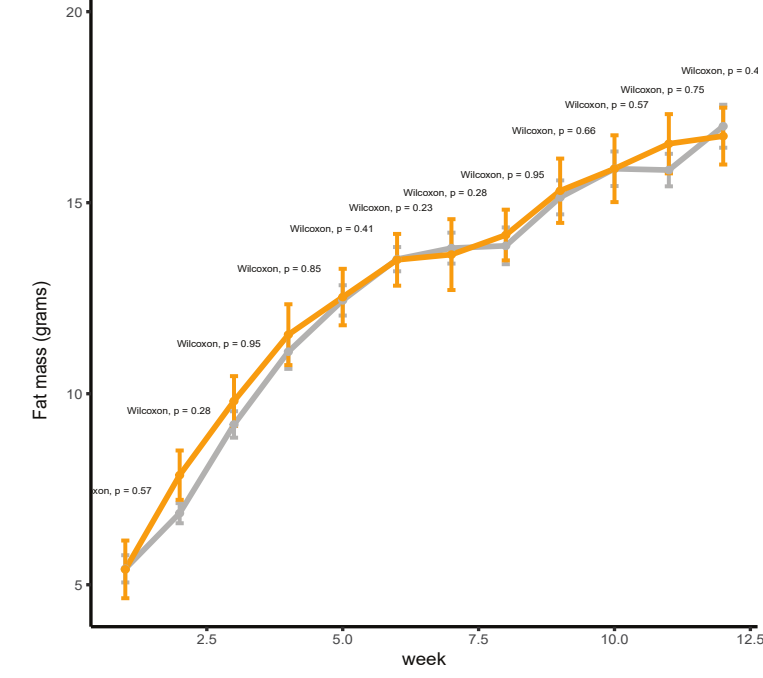

Supplement: Figure S7 — Body weight and fat mass measured over 12 weeks for mice expressing AAV-GFP (control, grey) or AAV-ITIH5 (orange). Experiments were conducted under standard chow diet conditions or a HF diet. Statistical significance between AAV-GFP and AAV-ITIH5 groups at each time point was determined using a Wilcoxon test, with p-values indicated above the respective data points. A statistically significant difference (p = 0.013, bold in red) was observed in the body weight of mice on the HF diet at week 5. [file mmc7.pdf]

S8

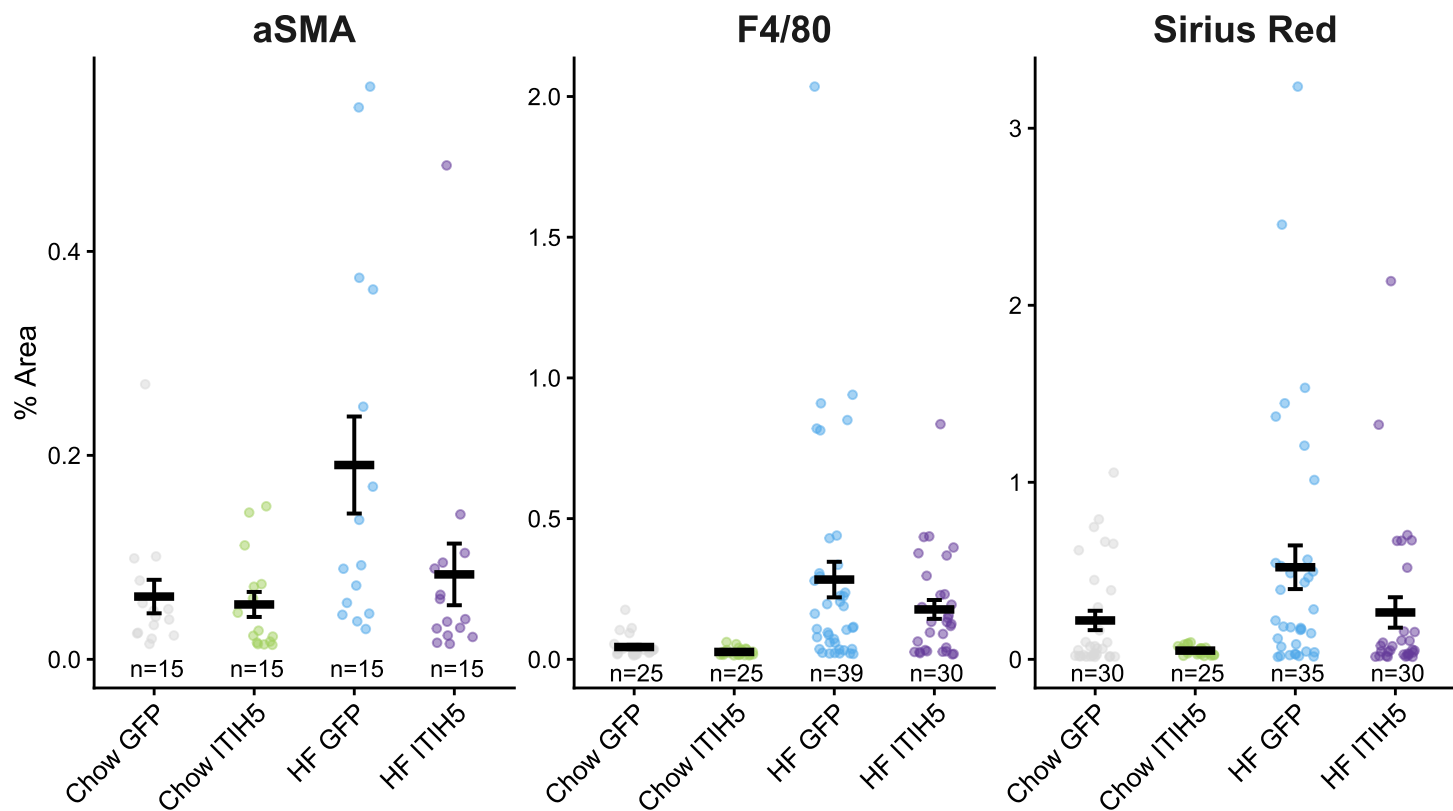

Supplement: Figure S8 — Percentage of positive stained area for aSMA, F4/80, and Sirius Red. Histological sections were evaluated from mice expressing either AAV-GFP or AAV-ITIH5, maintained on normal chow or a HF/HS diet. Image analysis and quantification were performed using ImageJ software. [file mmc8.pdf]

S9

LIVER

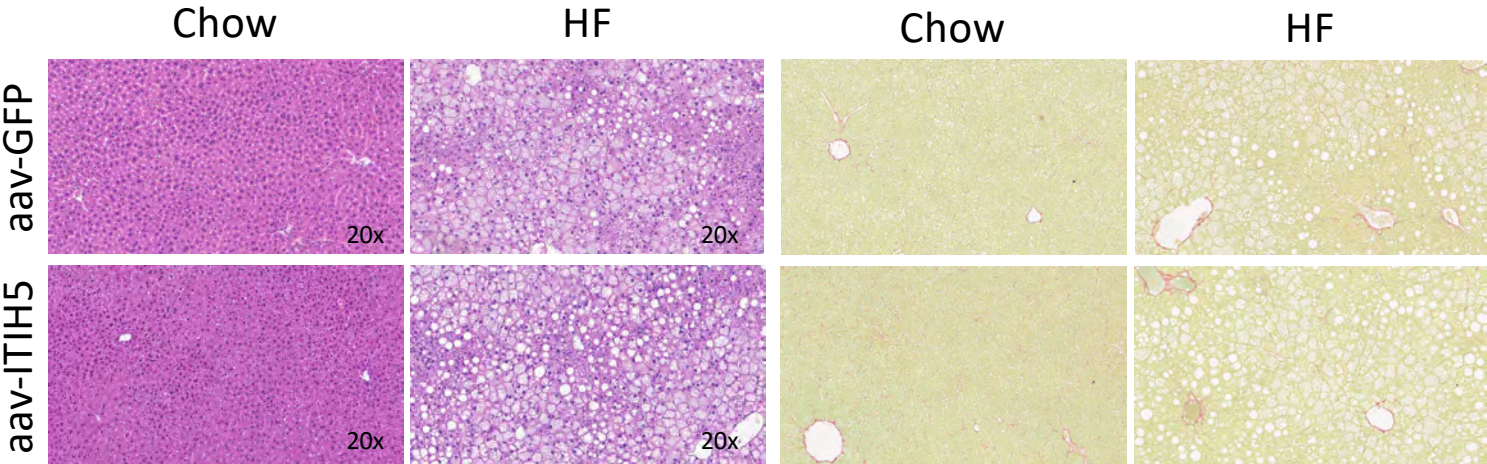

H&E

Sirius Red

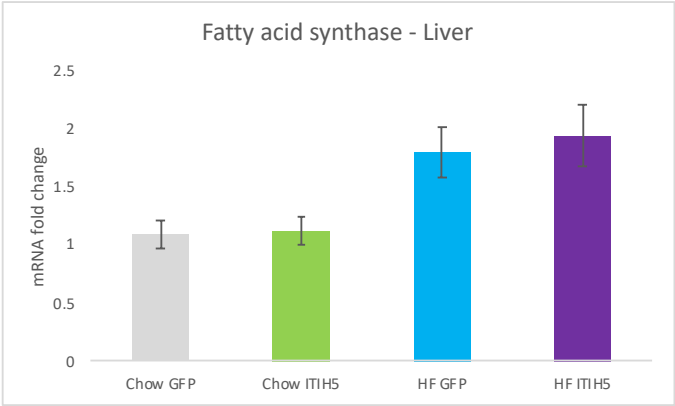

MUSCLE

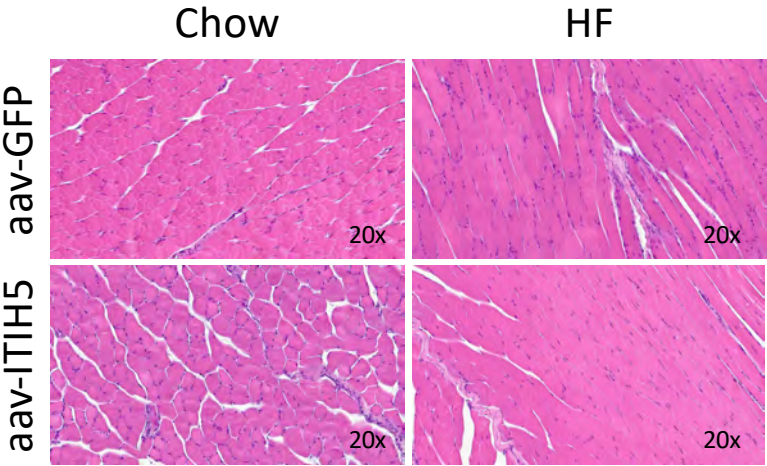

H&E

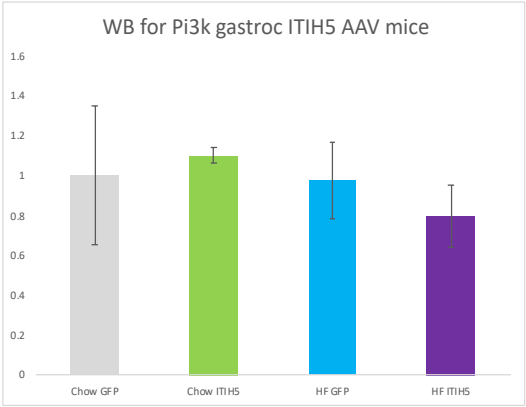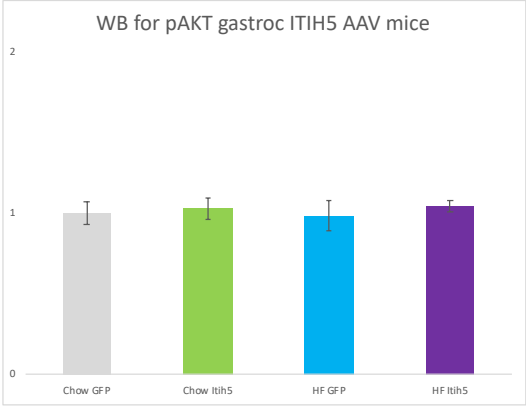

Supplement: Figure S9 — Histological and immunoblot comparisons of Liver (top) and muscle (bottom) between AAV-GFP and AAV-ITIH5 mice on normal chow and HF/HS diets. No notable differences were observed between groups. [file mmc9.pdf]
